# Production of fast-charge Zn-based aqueous batteries via interfacial adsorption of ion-oligomer complexes

**DOI:** 10.1038/s41467-022-29954-6

**Published:** 2022-04-27

**Authors:** Shuo Jin, Jiefu Yin, Xiaosi Gao, Arpita Sharma, Pengyu Chen, Shifeng Hong, Qing Zhao, Jingxu Zheng, Yue Deng, Yong Lak Joo, Lynden A. Archer

**Affiliations:** 1 Robert Frederick Smith School of Chemical and Biomolecular Engineering, Cornell University, Ithaca, NY 14853 United States; 2 Department of Materials Science and Engineering, Cornell University, Ithaca, NY 14853 United States

**Keywords:** Batteries, Batteries, Organic-inorganic nanostructures, Electrochemistry, Energy

## Abstract

Aqueous zinc batteries are attracting interest because of their potential for cost-effective and safe electricity storage. However, metallic zinc exhibits only moderate reversibility in aqueous electrolytes. To circumvent this issue, we study aqueous Zn batteries able to form nanometric interphases at the Zn metal/liquid electrolyte interface, composed of an ion-oligomer complex. In Zn||Zn symmetric cell studies, we report highly reversible cycling at high current densities and capacities (e.g., 160 mA cm^−2^; 2.6 mAh cm^−2^). By means of quartz-crystal microbalance, nuclear magnetic resonance, and voltammetry measurements we show that the interphase film exists in a dynamic equilibrium with oligomers dissolved in the electrolyte. The interphase strategy is applied to aqueous Zn||I_2_ and Zn||MnO_2_ cells that are charged/discharged for 12,000 cycles and 1000 cycles, respectively, at a current density of 160 mA cm^−2^ and capacity of approximately 0.85 mAh cm^−2^. Finally, we demonstrate that Zn||I_2_-carbon pouch cells (9 cm^2^ area) cycle stably and deliver a specific energy of 151 Wh/kg (based on the total mass of active materials in the electrode) at a charge current density of 56 mA cm^−2^.

## Abstract (web summary)

Aqueous zinc batteries attract interest because of their potential for cost-effective and safe electricity storage. Here, the authors develop an in situ formed ion-oligomer nanometric interphase strategy to enable fast-charge aqueous Zn cells.

## Introduction

Reversible electrodeposition of metals has recently reemerged as an area for fundamental and applications- oriented studies, reviving interest in rechargeable batteries that use metal anodes, such as Lithium (Li), sodium (Na), magnesium (Mg), calcium (Ca), zinc (Zn), and aluminum (Al)^1–4^. A large body of evidence now exists that uneven, mossy, and dendritic deposits, hinder reversibility in both expected (e.g., limiting battery lifetime through internal shorting) and un-expected (e.g., weakening the connections between deposits and the current collector to promote electronic isolation) ways. In either case, the result is that less of the active metal in the anode is available for charge storage, and consequently few, if any, of the metal anodes under consideration live up to their potential for achieving cost-effective and energy-dense storage^5,6^.

**Fig. 1:**
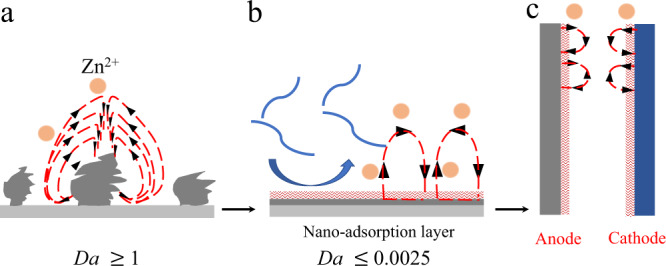
Ion movement mechanisms at a cation-selective interphase. **a** Schematic illustrating ion movement pattern at high current density. As the Damkohler number, *Da* ≥ 1, the body force exerted on electrolyte near a cation-selective interface drives a hydrodynamic instability known as “electroconvection” drive the growth of dendrite. The flat gray plate represents Zn plate. The dark gray agglomerates represent Zn dendrite. The yellow balls represent Zn^2+^. The red dashed lines and black arrows indicate the movement direction of cations under high *Da*. **b** However, as *Da* ≤ 0.0025, interfacial ion movement can be well regulated. The loose red layer represents nano-adsorption layer of ion-oligomer complex. The blue segments represent ion-oligomer complexes. The blue arrow represents the adsorption/desorption process of the ion-oligomer complex. The flat gray plate represents Zn plate. The dark gray flat plate represents uniform Zn deposits. The yellow balls represent Zn^2+^. The red dashed lines and black arrows indicate the movement direction of cations under low *Da*. **c** Illustration of the ion movement at the anode and cathode side with designed ion-oligomer electrolytes. The dark gray flat plate represents Zn anode side. The dark blue flat plate represents cathode side. The loose red layers represent nano-adsorption layer of ion-oligomer complex at both anode and cathode sides. The red dashed lines and black arrows indicate the movement direction of cations at electrode surface under low *Da*.

These findings have in turn renewed interest in understanding root causes and the fundamental transport and electrochemical processes that promote mossy deposition and dendrite formation over different ranges of current density, *i*^7,8^. In the low current density region—characterized as current densities well-below the classical diffusion-limited value, *i*_*L*_ = 4*FcD*_+_/*l*—classical transport-based theories that invoke hydrodynamic instability^8^ are not relevant. Here *i*_L_ is the limiting current; *D*_*+*_ is the cation diffusion coefficient; *c* the salt concentration in the electrolyte solution (assume to be dilute concentration: ≤0.05 M); *l* is the space between physically separated electrodes confined in the same cell; and *F* is Faraday constant. Thus, for *i* << *i*_L_, two processes are thought to be most responsible for non-uniform, wire-like and mossy deposition: (1) uneven electric field lines at irregular deposit features (e.g., bumps, edges, etc.), which promote growth of non-planar wire-like/mossy/dendritic structures^9^; and (2) uneven charge transport through interphases formed on the metal, leading to preferential nucleation and growth of metal deposits along particular directions. A serious consequence of the second process is that stripping of metal from underneath deposits coated by a non-uniform solid electrolyte interphase, can selectively remove portions of a non-planar deposit near its roots, which promotes breakage and electrochemical disconnection of the deposits (i.e., metal orphaning). This results in high anode irreversibility^10,11^, which typically means the reversible cycling of metal anodes is limited to a few cycles. All of these processes remain at high current density, *i* → *i*_L_, and are augmented by the hydrodynamic instability termed electroconvection, which is driven by an ion-depleted, extended space charge layer that extends away from the electrode surface into the bulk^12^. For fundamental reasons discussed elsewhere^13,14^, electroconvection favors localized deposition on a spectrum of length scales and recent studies, including numerical simulation and experimental observation^13–15^, show that strong “electroconvection” accelerates growth of non-planar deposit morphologies formed by other processes (Fig. 1a). A consequence is that operation of metal anode batteries is generally limited to the regime *i* << *i*_L_^4,16–22^, which is a barrier to their use in electric grid and fast-charging electrified transportation applications where long-term stability at high current densities is demanded.

Aqueous zinc batteries have recently been reported to provide excellent model systems for understanding interfacial processes in all batteries that employ metal anodes^23^. They also promise cost and performance advantages that are attractive from an applications perspective^24,25^. We note however that, notwithstanding the very high *D*_+_ values in aqueous electrolytes, the highest current density achieved in state-of-the art Zn cells^4^ are still substantially below *i*_L_ (≤40 mA cm^−2^). A number of strategies, including deployment of viscoelastic liquid electrolytes^7^ and field-induced crystallographic reorientation^8^ of Zn deposits have been reported to show potential for suppressing electroconvection and achieving primary smooth, uniform metal electrodeposition at high current densities. These effects are conventionally discussed in terms of coupling between electroconvection (which produces a spatially non-uniform cation flux at the electrode) and secondary flows produced by polymer elasticity or viscous fluid flow produced by electrode motion, either of which can produce counter flows that render the cation flux to the interface more uniform^26,27^. Local Joule heating has been reported as another important strategy to achieve smooth electrodeposition at high current density^28^. Results from linear stability analysis and direct numerical simulations from a number of groups, show that the Damkohler number *Da* ≡ *i*_0_/*i*_*L*_, the dimensionless ratio of exchange current *i*_0_ at the electrode to the diffusion-limited current *i*_*L*_ in the electrolyte determines the propensity for metals to deposit in largely planar/compact or non-planar/non-compact morphologies^14,26,27,29^. Here we make specific reference to findings from recent direct numerical simulations of coupled electro-hydrodynamics, transport, and interfacial reaction of metals at planar interfaces^14,27^, which show that as *Da* decreases, the lateral gradient of the surface potential creates a lateral ion flux that drives cations away from high cation concentration regions, meaning that the ion velocity (V_x_) parallel to the electrode surface increases, while the velocity perpendicular (V_y_) decreases (Fig. 1b). This finding is in qualitative accord with reports from direct tracer particle visualization experiments^15,30^, and can be interpreted more simply in terms of a competition between kinetic processes in the following manner: by allowing arriving metal ions sufficient time to redistribute at the electrode surface before they are reduced, low *Da* values are advantageous for obtaining a smooth electrodeposition morphology, irrespective of the current density. The fast reaction kinetics of metal electroreduction and large volume change at a metal anode required for practical battery operation are fundamental, un-resolved barriers for creating interphases that support sufficiently low *Da* at metal anodes in liquid electrolytes, as well as for maintaining such low *Da* values over long cell lifetimes and under conditions where the anode volume changes significantly during cycles of charge and discharge (Fig. 1b).

Here we report that dynamic, nanometer-thick, ion-oligomer interphases in situ formed on a Zn metal anode inside a single-cell battery provide a powerful and versatile approach for suppressing non-planar deposition by suppressing the full set of instabilities (chemical, morphological, and hydrodynamic) (Fig. 1b). The approach is stable during long-term operation (>10,000 cycles) of an unusually long-time scale (>1000 h). We show further that the spontaneously formed nanolayers enable highly reversible Zn electrodes at current densities as high as 160 mA cm^−2^ in aqueous electrolytes, which is at the same order of magnitude of the limiting current density (*i*_L_ ≈ 10^^2^ mA cm^−2^), and about four times higher than the highest values reported for aqueous Zn electrodes. When paired with conversion cathodes such as I_2_/activated carbon, we demonstrate that the dynamic ion-oligomer complexes enable battery cells, pseudocapacitor-like lifetimes (>12,000 charge/discharge cycles) at high active material utilization. In addition, fast-charge pouch cell batteries employing a 20-μm thin Zn anode are demonstrated to run for over 100 cycles at a charge current density of 56 mA cm^−2^. Even without efforts to optimize their performance, the pouch cells achieve specific energy as high as 555 Wh kg^−1^_AC_ (151 Wh kg^−1^_Zn+AC_), illustrating the scalability of the ion-oligomer electrolyte concept. As an extended example, we investigate Zn||MnO_2_ aqueous cells and find that they also achieve long-term cycling performance (>1000 charge/discharge cycles) at a charge current density of 160 mA cm^−2^, meaning the reported ion-oligomer complexes are quite versatile for both anode and cathode sides (Fig. 1c). We also explore the possibility to apply the in situ formed ion-oligomer nanometric interphase strategy in a non-aqueous electrochemical energy storage system such as an Li||I_2_ cell where we demonstrate reversibly battery operation at a current density of 40 mA cm^−2^.

## Results

As a first step to identifying a general strategy for achieving high metal anode reversibility, we speculate that a conformal, interfacial nanolayer spontaneously formed on a metal electrode by reversible adsorption of poly-/oligo-ethers could simultaneously block electroreduction sites—lowering *i*_0_—and providing a “self-healing” (polymer layer can be spontaneously reformed during metal strip and deposit process) characteristic stemming from dynamic polymer adsorption/desorption processes at the electrolyte/electrode interface. To evaluate this hypothesis, we take advantage of the following three well-known effects to create and study adsorption of Zn^2+^-halide ion-oligomer complex at metal substrates: (i) high affinity of metal cations for the lone electron pairs on ether oxygens in poly-ethers; (ii) high surface affinity of halide ions for metal substrates, which has been attributed to their ability to form interfacial halide-metal covalent-like bonds^31^; and (iii) spontaneous dynamic adsorption/desorption of dissolved polyether ion complexes to the electrode surface. Thus, a well-designed halide ion-oligoether complex, provides a possible mechanism for reversibly drawing the oligomer out of solution and onto the metal surface, and anchoring it there using strong interfacial bonds between halide and metal. The bonding between Zn^2+^ and oligomer increases the de-solvation energy of Zn-ion at the electrode, lowering *i*_0_ for the interfacial reduction of Zn^2+^^32,33^. On this basis we speculate that an effective polymer adsorption layer requires a balance between two polymer solution effects: ion-oligomer complex formation and interfacial complex adsorption. The hydrated oligomer must first bond with the initial complexes of Zn^2+^-halide ions in the bulk electrolyte to create the complex, while remaining soluble. The second requirement is that the Zn^2+^-halide ion-oligomer complex dynamically adsorbs onto the surface by the process mentioned above.

### Zn^2+^-halide ion-oligomer design and adsorption

We designed and studied the structure of ion-oligomer complexes in aqueous electrolytes formed between Zn^2+^-halide ion complexes and a polyethylene oxide (PEO) oligomer of average molecular weight 300 Da. Aqueous electrolytes solutions based on three zinc halide salts (ZnX_2_ (X = Cl, Br, I)) were prepared and the results compared with an aqueous ZnSO_4_ reference electrolyte solution. The structure of the solvated Zn halide complexes deduced from Raman vibrational spectroscopy of the electrolytes (Supplementary Fig. 1) are consistent with previous reports^34–36^ and show that structure of the initial ion complexes formed by Zn salts in aqueous media varies substantially from salt to salt. For ZnSO_4_ electrolyte, octahedral [Zn(H_2_O)_6_]^2+^ is the dominant ionic complex in solution (Supplementary Fig. 2a); in ZnCl_2_ electrolyte, tetrahedral [ZnCl_2_(H_2_O)_2_], in which Zn ions bond with two water molecules and two chloride ions, are the main ionic states (Supplementary Fig. 2b); in ZnI_2_ electrolyte, in addition to the stable complexes [ZnI_4_]^2−^ (in which Zn only bonds with iodide ions in aqueous media), several unstable complexes [ZnI_3_]^−^, [ZnI_2_] and [ZnI]^+^ are also present in large proportions (Supplementary Fig. 2c); in ZnBr_2_ electrolyte, the ion complexes states combine the structure of ZnCl_2_ and ZnI_2_ electrolyte, [ZnBr_2_(H_2_O)_2_], [ZnBr_4_]^2−^, [ZnBr_3_]^−^, [ZnBr_2_] and [ZnBr]^+^ coexist and the ratio of these ion complexes varies as the concentration of ZnBr_2_ change (Supplementary Fig. 2d). The different initial ion complex states result from the different anion steric structures (SO_4_^2−^ and halide ions) and electronegativity (Cl, Br, I). The LUMO potential of all solvation ions was given by simulation (Supplementary Fig. 3), which explains the different reaction kinetics (exchange current) in the following sections.

Long-chain PEO (M_W_ > 3000) shows a great potential to regulate Zn electrodeposition due to several reasons^37^: (1) PEO can increase the viscosity of the electrolyte that has a great influence on the ion mobility; (2) due to the interfacial electrode adsorption of PEO, visualization experiments show that the ion velocity V_x_ highly suppressed while V_y_ increases (Fig. 1b) that may result from the relaxation and contraction of long polymer chain. On the basis of these results, we choose to study polyethylene glycol with a molecular weight of 300 g mol^−1^ (PEG300). This polymer is of interest because it is easily dissolved in aqueous Zn salt electrolytes to form ion-oligomer complexes. We observed different ion-oligomer complex structures when PEG300 is introduced to the four aqueous Zn salt-based electrolytes.

**Fig. 2:**
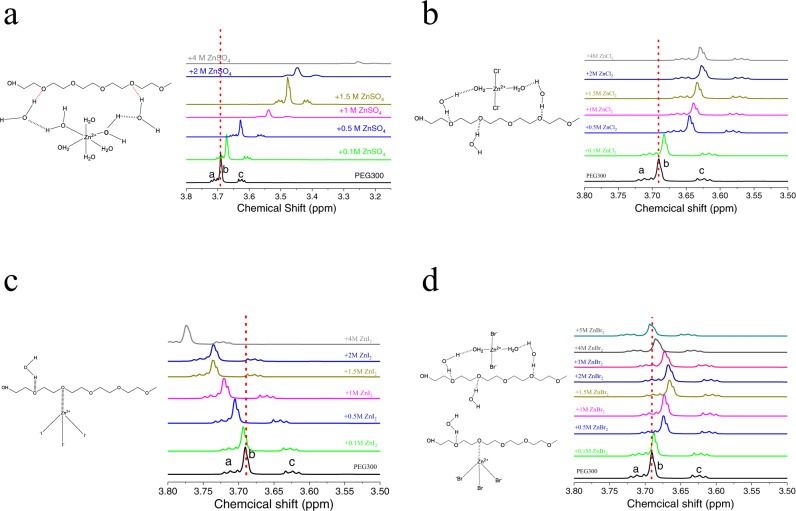
Ion-oligomer complex formed in ZnSO_4_ and ZnX_2_ (X = Cl, Br, I) aqueous electrolytes. Model for bonding between PEG and Zn ion complexes (left) and ^1^H nuclear magnetic resonance (NMR) analysis (right) in various concentrations of **a** ZnSO_4_, **b** ZnCl_2_, **c** ZnI_2_, and **d** ZnBr_2_ electrolytes at containing a fixed concentration (5 wt%) of polyethylene glycol (PEG300) oligomer.

**Fig. 3:**
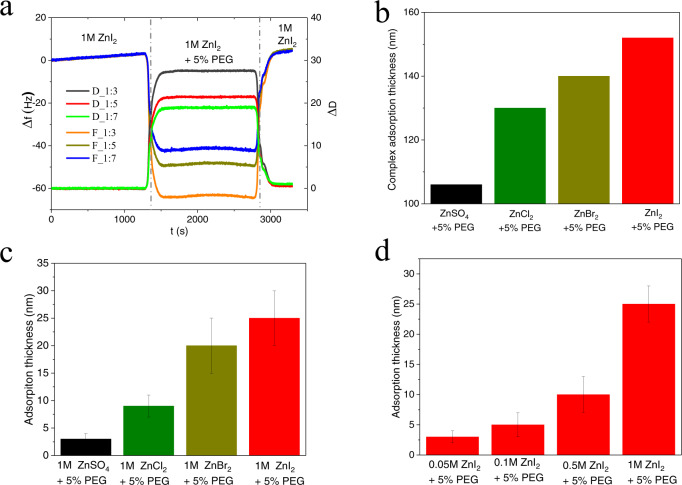
Interfacial adsorption thickness of 1 M ZnSO_4_ and 1 M ZnX_2_ (X = Cl, Br, I) aqueous electrolytes containing oligomeric polyethylene glycol (PEG 300 g mol^−1^) as a soluble additive. **a** QCM analysis of 1 M ZnI_2_ and 1 M ZnI_2_ + 5% PEG electrolytes. ∆f is the frequency change and ∆D is dissipation change. F_1:X (X = 3, 5, 7) represent different harmonics and D_1:X (X = 3, 5, 7) represent different dissipation values. **b** Ion-oligomer adsorbate thickness extracted from (**a**). **c** Adsorption thickness of different Zn Salts + 5% PEG using ellipsometer. The error bars (standard deviation) were calculated based on at least three sets of data. **d** Adsorption thickness of different concentrations of ZnI_2_ + 5% PEG using ellipsometer. The error bars (standard deviation) were calculated based on at least three sets of data.

Nuclear magnetic resonance (NMR) was used to first understand the ion-oligomer complexes formation in aqueous electrolytes containing Zn salts and PEG300. It is known that the de-shielding effect results in the left shift of ^1^H peak in NMR spectra, therefore, the ^1^H peak in the PEG backbone (Supplementary Fig. 4) reflects the complexes’ stability of PEG300, Zn^2+^, and halide ions. In 5% PEG300 + ZnSO_4_ electrolyte, the ^1^H peaks of PEG300 shift to the far right, indicating the strong shielding effect here, which increases as the concentration of ZnSO_4_ increases (Fig. 2a). We assume that it’s the hydrogen bond between Zinc solvation ions, water molecules, and PEG300 that results in this kind of effect, and further predicts the bond model (Fig. 2a). To evaluate our hypothesis, Fourier-transform infrared spectroscopy (FTIR) and ab initio calculations are used to understand the hydrogen bonding effect. In FTIR (Supplementary Figs. 5–7), the ratio between the absorbance intensity of Peak c and Peak d can reflect the level of hydrogen bond cooperativity^38,39^. The value of Peak c/Peak d decreases as 5% PEG is added into the DI water, meaning that much higher hydrogen bonds cooperativity and the interaction between water molecules and PEG is stronger (Supplementary Fig. 6). 1 M ZnSO_4_ salts can further decrease the Peak c/Peak d value, which can be explained by the formation of Zn solvation ions (Supplementary Fig. 6). An intense decrease of Peak c/Peak d value is observed in the 5% PEG300 + 1 M ZnSO_4_ electrolyte, verifying that hydrogen bond is the main factor that facilitates the PEG300–H_2_O–[Zn(H_2_O)_6_]^2+^ complex (Supplementary Fig. 6). Ab initio calculations further confirms the complex formation model (Supplementary Table 1). However, a different shift trend of the PEG300 ^1^H peak is observed in Zn halide electrolytes. For ZnCl_2_ + 5% PEG electrolytes (Fig. 2b), the shielding effect (peak shift) is not as strong as that of ZnSO_4_ electrolyte, indicating lower hydrogen bond cooperativity that can be further confirmed by the increase of Peak c/Peak d value (Supplementary Fig. 6). Nevertheless, the reverse shifting trend (de-shielding effect) in ZnI_2_ + 5% PEG electrolytes indicates a different bonding effect between PEG and the unstable complex [ZnI_3_]−, [ZnI_2_] and [ZnI]^+^, in which no water molecules take part in the ion-oligomer complex formation (Fig. 2c) and the Peak c/Peak d value highly increases (Supplementary Fig. 7). Such a behavior can be explained by the lower electronegativity and higher polarization of iodine atoms, resulting in a more uniform electron distribution and less partial charge at both Zn and I. Compared to the highly polarized Zn-Cl bond, the [ZnI_x_]^2−x^ complex is less prone to bond with free water (a strong dipole), but prefers bonding with the oxygen atom on PEG, whose partial charge is stabilized by the carbon backbone. The above mechanism is verified in ab initio simulations (Supplementary Tables 2 and 3). For ZnBr_2_ + 5% PEG electrolytes, shielding effect is observed initially (shift to the right) and then overwhelmed by the de-shielding effect (shift back to the left), indicating the ion-oligomer formation effect changes as the concentration of ZnBr_2_ increases (Fig. 2d). Bromine has an intermediate electronegativity and polarization compared to chlorine or iodine, and thus demonstrates a mixed behavior of both. On the basis of the ^1^H spectrum of NMR results (Fig. 2), hydrogen bond peaks of FTIR (Supplementary Figs. 6 and 7), we deducted the complex structure for the Zn halide + PEG electrolytes: (1) PEG-H_2_O-[ZnCl_2_(H_2_O)_2_] complex in ZnCl_2_ electrolyte (Fig. 2b); (2) PEG-[ZnI_3_]^−^, [ZnI_2_] and [ZnI]^+^ complexes in ZnI_2_ electrolyte (Fig. 2c); (3) PEG- H_2_O-[ZnBr_2_(H_2_O)_2_], PEG-[ZnBr_3_]^-^, [ZnBr_2_] and [ZnBr]^+^ complexes in ZnBr_2_ electrolyte (Fig. 2d). All these models are consistent with the ab initio calculations (Supplementary Figs. 8a, b and 9).

The influence of the ion-oligomer complexes on interfacial polymer adsorption was studied using a quartz-crystal microbalance (QCM), ellipsometry, and electrochemical impedance spectroscopy (EIS). QCM (Fig. 3a, b and Supplementary Fig. 10a–d) is very sensitive to the interfacial polymer adsorption process and can be used to characterize the amount, thickness, reversibility, and interfacial physical properties of the adsorbed polymer layer. We employed three electrolyte flowing steps protocol. First, flowing the blank 1 M ZnSO_4_ aqueous electrolyte solution or Zn halide electrolyte solution to establish the effect of salts adsorption on the frequency shift. Second, 5% PEG300 + 1 M ZnSO_4_ or Zn halide electrolytes flow through QCM sensor, we can observe the frequency change (∆*f*) and dissipation change (∆*D*) (“dissipation” is the sum of all energy losses in the flowing system per oscillation cycle) which can be related to physical properties of the polymer adsorbate (including thickness, viscosity, and modulus) as well as the viscosity increment produced in the electrolyte bulk. Finally, after the stabilization of the second step, we re-flow the blank 1 M ZnSO_4_ aqueous electrolyte solution or Zn halide electrolyte solution. In this step, if the frequency returns to the value in the first step, the polymer adsorption in the second step is considered reversible.

Analysis of the QCM experiments revealed a number of important features of the interphases formed by polymer adsorption: (1) Ion-oligomer adsorption is a reversible process since the frequency and dissipation values plummet to zero when the flowing fluid is switched back to the initial solution (1 M ZnSO_4_ or 1 M ZnX_2_), not containing polymer; (2) as shown in Fig. 3b and Supplementary Fig. 10e, the average thickness is related to the interfacial coverage, meaning the thicker the adsorbate layer, the greater the coverage. The designed complex of Zn^2+^-halide ions-PEG300 shows larger interfacial coverage than PEG-[Zn(H2O)_6_]^2−^ complex, which we believe reflects the strong interfacial specific adsorption of halide ions^33^; (3) however, the interfacial viscosity (Supplementary Fig. 10f) of these four kinds of complexes does not vary greatly, implying that the interfacial viscosity is not the main source of the change of interfacial properties, which disagrees with previous reports^7^; (4) the ion-oligomer adsorption layer is viscoelastic in nature since the shift in different harmonics do not overlap (F_1:3, F_1:5, F_1:7, the number 3, 5, 7 represent the third, fifth and seventh harmonics) and the dissipation values (D_1:3, D_1:5, D_1:7, the number 3, 5, 7 represent the dissipation change for different harmonics) are high (>1 × 10^−6^). Direct measurement of ion-oligomer complex layer features was performed using an ALPHA-SE Ellipsometer (Supplementary Fig. 11). These measurements confirm the strong interfacial adsorption of Zn^2+^-halide ions-oligomer complex (Fig. 3c, d). Based on the QCM and ellipsometer results, the adsorbate thickness of the designed ion-oligomer complex varies from 2 to 150 nm, depending on the anion chemistry and polymer concentration. Hence, we can conclude that the influence of the adsorption layer is concentrated in a nanometer-thick interphase zone near the electrode surface.

**Fig. 4:**
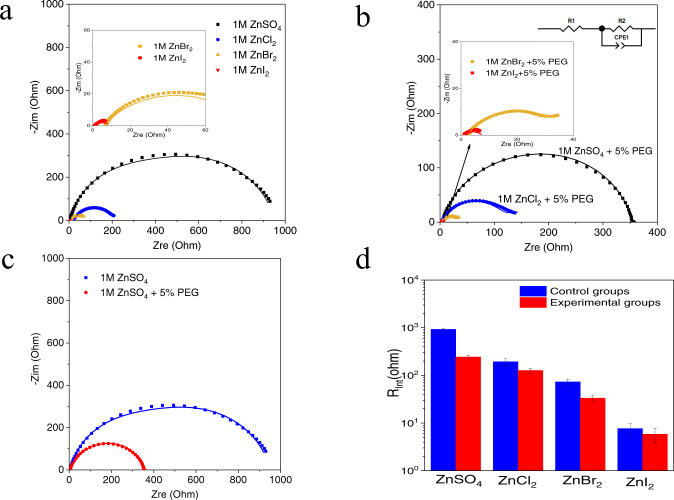
Interfacial resistance of 1 M ZnSO_4_ and 1 M ZnX_2_ (X = Cl, Br, I) aqueous electrolytes containing 5 wt% PEG300. Electrochemical impedance spectroscopy (EIS) of (**a**) 1 M ZnSO_4_ and ZnX_2_ (X = Cl, Br, I) aqueous electrolytes; (**b**) 1 M ZnSO_4_ and ZnX_2_ aqueous electrolytes containing 5 wt% PEG300. Inset reports results from EIS analysis of Zn in a 1 M ZnI_2_ + 5% PEG300 electrolyte. The measurements were all performed in Zn||Zn symmetric cells; the raw impedance values are reported as symbols in the plots. The lines through the data are best fit curves obtained using the equivalent circuit model depicted on the top right of (**b**); (**c**) 1 M ZnSO_4_ with/without 5% PEG300. (**d**) Interfacial resistance (Rint) obtained by fitting the data in (**a**–**c**) using the equivalent circuit model specified in (**b**) (top right). Control groups and experimental groups are terminology used throughout the paper to reflect results obtained, respectively, using electrolytes without and with 5% PEG300. The error bars (standard deviations) were calculated based on at least two sets of data.

EIS experiments can also provide complementary insights into the dynamics of an interfacial nanolayer composed of the designed Zn^2+^-halide-PEG300 complex. Figure 4a–c, Supplementary Fig. 12a–d and Supplementary Table 4 show that much lower interfacial impedance are observed in electrolytes containing ZnI_2_ and that these values are reduced slightly in electrolytes containing PEG oligomer additives. These results are consistent with our previous report^33^, which showed the strong interfacial adsorption of unstable iodide and bromide ion complexes facilitate interfacial ion transport. Our earlier results showed further that these complexes are stabilized by adsorption to PEG oligomers, which we believe results in the reduced interfacial impedance observed (Fig. 4d).

### Effect of the ion-oligomer complex layer on the Zn electrodeposition

**Fig. 5:**
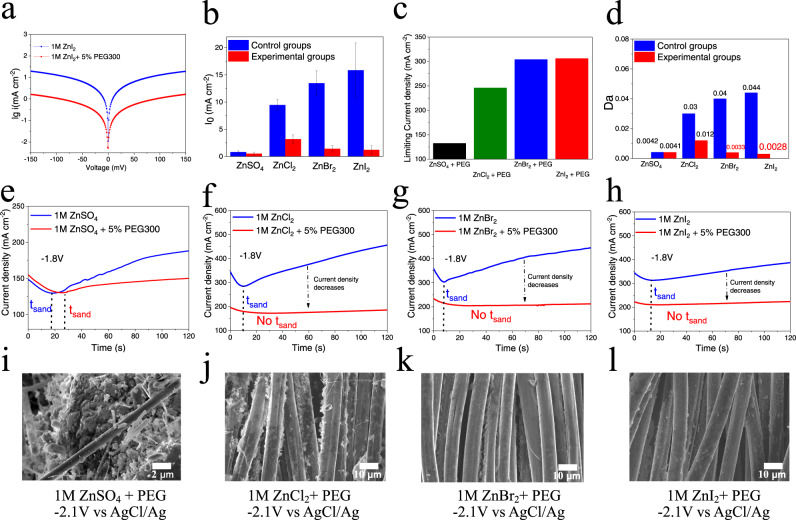
Electrochemical characteristics of 1 M ZnSO_4_ and 1 M ZnX_2_ (X = Cl, Br, I) aqueous electrolytes containing 5 wt% PEG300. **a** Tafel plots of 1 M ZnI_2_ with/without 5% PEG300, derived from cyclic voltammetry measurements. **b** Exchange current densities (*i*_0_) calculated using the Tafel plots. The error bars (standard deviation) were calculated based on at least two sets of data. **c** Limiting current density extracted from the current–voltage (i–v) curve. **d** Damkohler number Da = Exchange current/Limiting current. **e**–**h** Current-time (i–t) curve at constant voltage, –1.8 V vs AgCl/Ag(V). The black dashed line represents sand time points t_sand_. **i**–**l** Electrodeposition morphology at the over-limiting current region, –2.1 V vs AgCl/Ag(V) for 15 s. The deposition substrate is three-dimensional (3D) carbon cloth.

Figure 5 reports the effect of the ion-oligomer complex layer on the Zn electrodeposition process. First, we interrogate the effect of the adsorbed polymer on the exchange current density (Fig. 5a, b and Supplementary Fig. 13). In all electrolytes studied, the PEG is seen to markedly lower *i*_0_ (Fig. 5b), which we attribute to ion-oligomer adsorption, which can increase the de-solvation energy of Zn^2+^ by the aforementioned mechanism. Next, limiting current densities extracted from the current–voltage (i–v) curve (Supplementary Fig. 14) can further reflect the interfacial ion diffusion kinetics. Zn halide + PEG electrolytes show much higher limiting current densities than ZnSO_4_ + PEG electrolytes (Fig. 5c), which results from the strong interfacial adsorption of Zn-halide ion complexes (such as [ZnI_3_]^−^)^33^, further supporting the speculation that halide ions can improve the interfacial adsorption of ion-oligomer complex. Based on these results, we can combine the influence of reaction kinetics and diffusion kinetics by *Da* (*Da* ≡ *i*_0_/*i*_*L*_). Previous numerical simulations^14,27^ show that when *Da* reaches a critical low value of 0.0025, a uniform ion flux parallels (V_y_) at the electrode surface can be achieved. As Shown in Fig. 5d, *Da* values below this theoretical threshold are observed in ZnBr_2_ + PEG300 (*Da* ≈ 0.0033) and ZnI_2_+ PEG300 (*Da* ≈ 0.0028) electrolytes. Chronoamperometry experiments were performed to more directly correlate the transient current response and electrodeposition morphology under conditions approaching the transport limiting current range. The inflection in the current versus time (i–t) plots for the pure Zn salt and ZnSO_4_ + PEG electrolytes is associated with the Sands time (Fig. 5e), indicative of the time-scale required for the space charge to develop and mixing due to formation of electroconvection rolls to begin^8^. The inflection disappears in ZnX_2_ (X = Cl, Br, I) + PEG electrolytes (Fig. 5f–h). Significantly, smooth and uniform Zinc deposits were observed in the ZnBr_2_+ PEG and ZnI_2_ + PEG electrolytes (Fig. 5i–l), even at current densities in the classical over-limiting electrokinetic regime region (–2.1 V vs AgCl/Ag). Our finding that the Zn electrodeposit morphology is uniform even at the high voltages used in the study implies that it should be possible to create stable Zn batteries able to operate at high current densities as discussed in the following section.

### Assembly and testing of aqueous Zn metal lab-scale cells

**Fig. 6:**
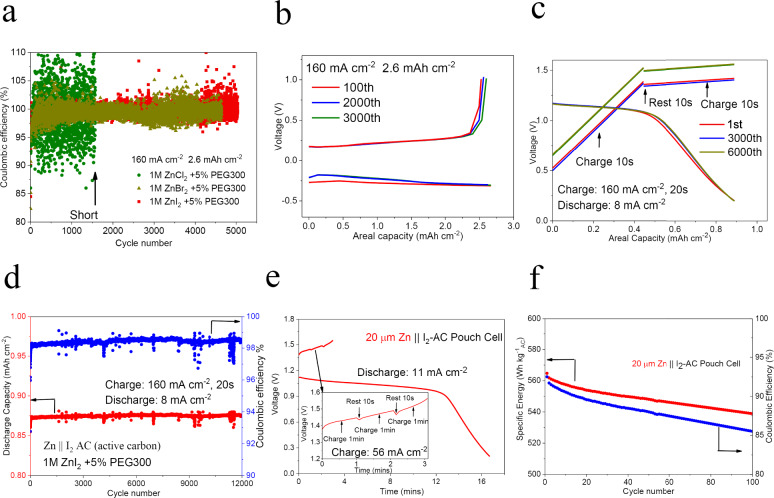
Galvanostatic cycling behavior of Zn anodes and fast-charge aqueous batteries. **a** Coulombic efficiency measured in Zn plate-strip experiments in Zn||carbon matrix half cells in 1 M ZnX_2_ (X = Cl, Br, I) +5 wt% PEG300 electrolytes at 160 and 2.6 mAh cm^−2^. **b** Voltage–capacity curve for the corresponding Zn||carbon matrix half-cell in 1 M ZnI_2_+5 wt% PEG300. **c** The voltage–capacity curve for the fast-charge aqueous Zn||I_2_ Active carbon (AC) full cell in different cycles. Charge process: charging at 160 mA cm^−2^ for 10 s, resting for 10 s, charging another 10 s at 160 mA cm^−2^; discharge process: discharging at 8 mA cm^−2^. The time step of recording is 10 s. **d** Discharge capacity and Coulombic efficiency of fast-charge aqueous Zn||I_2_ (AC) full cell over galvanostatic cycling. **e** The time–voltage curve in the first cycle of the fast-charge Zn(20 µm)||I_2_ (AC) pouch cell. Charge process: charging at 56 mA cm^−2^ for 1 min, resting for 10 s, and repeats three times; discharge process: discharging at 11 mA cm^−2^. **f** The specific energy (based on the mass of AC) and Coulombic efficiency of fast-charge aqueous Zn||I_2_ (AC) pouch cell.

To evaluate the reversibility of Zn deposits in our designed ion-oligomer electrolytes at the high current density, we measured the electrochemical cycling performance of Zn anodes in Zn||carbon cloth half-cell. The plating/stripping Coulombic efficiency (CE %) of Zn deposits in each cycle reveals the ratio between the amount of metal that can be stripped and the amount that is plated in the previous cycle. Figure 6a and Supplementary Fig. 15a show the reversible Coulombic efficiency (CE %) for a long time cycling at current density 160 mA cm^−2^ and areal capacity 2.6 and 1.1 mAh cm^−2^. Compared with ZnI_2_ (Supplementary Fig. 15b), ZnSO_4_ + PEG300 (Supplementary Fig. 16a) and ZnCl_2_ + PEG300 electrolytes (Fig. 6a), ZnBr_2_ + PEG300 and ZnI_2_ + PEG300 electrolytes (Supplementary Figs. 16b, 17, and 18), show more stable and higher CE (99.2% for ZnBr_2_ + PEG and 99.5% for ZnI_2_ + PEG, respectively) and better reversible cycling performance (5000 cycles at 2.6 mAh cm^−2^ and 8000 cycles at 1.1 mAh cm^−2^). It is worth noting that the stable reversible cycling performance at a current density of 160 mA cm^−2^ was achieved on a Zn anode (Fig. 6).

Benefiting from the long cycle life of the Zn anode at high current density (160 mA cm^−2^), fast-charge aqueous batteries can be produced using ZnI_2_ + PEG electrolytes. For ZnI_2_ + PEG electrolyte, we choose active carbon (AC) as cathode, which can provide sufficient adsorption site^33^ for the oxidation product I_3_^−^ (3I^−^ → I_3_^−^ + 2e^−^). Here, to compensate for the relatively low reaction rate at cathode side, we designed an intermittent charging method for Zn||I_2_(AC) full cell: charging 10 s at 160 mA cm^−2^, resting 10 s to enable the interfacial ion equilibrium and then charging another 10 s. As shown in Fig. 6c, voltage increase can be observed in both charging stages, while the voltage drop during the 10-s rest is minimal, demonstrating stable charging behavior. During the discharging process, a stable discharge plateau at 8 mA cm^−2^ proves the effectiveness of this charge process and the stability of the electrode at ZnI_2_ + PEG electrolytes. Significantly, Zn||I_2_(AC) full cell with 1 M ZnI_2_ + 5% PEG electrolytes shows stable cycle performance: at a charge current density of 160 mA cm^−2^ and discharge current density of 8 mA cm^−2^, we achieved an areal capacity of 0.88 mAh cm^−2^ over 12,000 cycles (Fig. 6d), benefiting from the stable Zn electrodeposition and intermittent charging method (Supplementary Fig. 19). Importantly, the designed 1 M ZnI_2_ + 5% PEG electrolytes and intermittent charging method can also be applied to pouch cells with 20-μm thick zinc foil (Supplementary Fig. 20). By using ZnI_2_ + 5% PEG electrolytes and 20 μm Zn foil, Zn||I_2_ (AC) pouch cell can be charged at a high current density of 56 mA cm^−2^ and run stably over 100 cycles. For pouch cell, we employed a similar intermittent charging method: charge the pouch cell for 1 min at 56 mA cm^−2^, then rest for 10 s, and repeat three times (Fig. 6e). Similar to coin cell, a distinct discharge plateau can be observed with discharge current density of 11 mA cm^−2^, proving the effectiveness of our ion-oligomer electrolyte system and intermittent charging method. Notably, a specific energy of 555 Wh kg^−1^_AC_ at a current of 0.506 A can be achieved based on the mass of AC, and 151 Wh kg^−1^ can be retained considering the total mass of AC and 20 µm Zn anode (Fig. 6f).

### Investigation on the interfacial ion movement mechanism

On the basis of previous simulation results^14,26,27^ and our experimental results, we investigated the interfacial ion movement mechanism at the ion-oligomer nanolayer adsorbed on the electrode. Without the ion-oligomer adsorption layer, the high Da value leads to dendrite growth by physical and hydrodynamic instability at high current densities (Fig. 1a). Simulation results show that a viscoelastic polymer coating layer^14,26,27^ can regulate the interfacial ion movement by lowering *Da* (≤0.0025). With a small *Da*, the interfacial ion movement velocity (V_x_ and V_y_) can be regulated as mentioned before and smooth electrodeposition can be achieved. However, considering the mechanical stability of the viscoelastic polymer coating layer, the stagnant coating layer is not suitable for long-term battery cycling. Here, the small *Da* of “self-healing” dynamic ion-oligomer adsorption layer in ZnBr_2_ + PEG300 (*Da* ≈ 0.0033) and ZnI2+ PEG300 (*Da* ≈ 0.0028) electrolytes indicate the interfacial ion movement regulation effect also exists here (Fig. 1b). To prove such an effect, we firstly obtained different i–v curves at different concentrations in these four Zn salts (Supplementary Fig. 14). The voltage stability window ΔV extracted from the i–v curve is consistent with the bonding effect and the intensity of the ion-oligomer adsorption layer, proving that the ion movement is influenced by the ion-oligomer adsorption layer (detailed explanation see Supplementary Note 1).

**Fig. 7:**
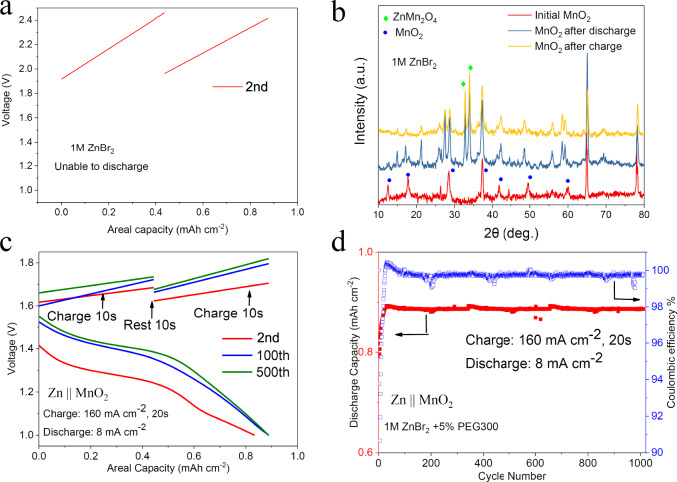
Fast-charge process at Zn battery cathodes with different chemistries. **a** The voltage–capacity curve of fast-charge aqueous Zn||MnO_2_ full cell at 1 M ZnBr_2_ electrolyte solution. **b** Ex situ XRD measurements of MnO_2_ before cycling and after fully discharge/charge process. The cell was disassembled after one full cycle of charge and discharge then the electrode collected. **c** The voltage–capacity curve for a fast-charge aqueous Zn||MnO_2_ full cell battery utilizing a 1 M ZnBr_2_ +5 wt% PEG electrolyte. The charging protocol is as follows: charge cell at 160 mA cm^−2^ for 10 s; rest for 10 s, charge cell at 160 mA cm^−2^ for another 10 s; discharge cell at 8 mA cm^−2^. **d** Discharge capacity and Coulombic efficiency of fast-charge Zn||MnO_2_ full cells containing 1 M ZnBr_2_ +5 wt% PEG electrolyte.

Furthermore, if the ion movement regulation effect can work well for the Zn anode, the same kind of influence can also work on the intercalation/ de-intercalation cathode at high current density (Fig. 1c). Generally, high current density leads to much more serious problems including electrochemical and hydrodynamic instability for intercalation/de-intercalation cathode materials. Hence, we choose layered MnO_2_ cathode^40^ (Supplementary Fig. 21) and ZnBr_2_ + PEG electrolyte here. The same intermittent charging method for Zn||MnO_2_ full cell is applied here: charging 10 s at 160 mA cm^−2^, resting 10 s, and then charging another 10 s. The voltage–capacity curve and X-ray diffraction experiments (Fig. 7a, b) show that without regulation of polymer, Zn^2+^ is hard to be extracted from MnO_2_ cathode at super high current density, which should be the main reason that the MnO_2_ is unable to discharge at blank ZnBr_2_ electrolyte. However, as shown in Fig. 7c, a slight voltage increase can be observed in both charging stages, demonstrating a potential voltage plateau, while the voltage drop during the 10-s rest is minimal, demonstrating stability. On the other hand, during the discharging process, a stable discharge plateau at 8 mA cm^−2^ proves the ion movement at cathode sides can also be regulated by the ion-oligomer adsorption layer (Supplementary Fig. 22 and Fig. 7d). Considering the simplicity and effectiveness of the approach, we wondered whether it could be used to enable fast charging of non-aqueous Li-based batteries. Supplementary Fig. 23 reports results showing that the concept is effective for lithium-iodine cells. Specifically, we find that Li||I_2_ cell that use LiI and 1% PEG300 in a DOL-based electrolyte, can be charged at current densities as high as 40 mA cm^−2^ (Supplementary Fig. 23a). Compared with the electrolyte systems without PEG300 additive (Supplementary Fig. 23b), the LiI + 1% PEG additive can well stabilize the lithium electrodeposition and lead to high Coulombic efficiency (93%) in Li||I_2_ cell over 200 cycles (Supplementary Fig. 23c). However, the results reported still need to be further optimized the ion-oligomer complex and electrolyte solvent for Li. Taken together with the more in-depth results for the Zn system, the Li results nonetheless lead us to conclude that the interfacial ion regulation mechanism achieved by the ion-oligomer nano-adsorption layer is quite versatile.

In summary, we report that controlling the Zn electrodeposition process at high current density (e.g., 160 mA cm^−2^) can be achieved by an interfacial ion-oligomer interphase with small Damkohler number *Da*. By designing the Zn^2+^-halide ion-PEG complex, reversible interfacial ion-oligomer adsorption can meet both the experimental and theoretical requirements (small *Da*). Small *Da* indicates that the ion-oligomer adsorption layer can effectively regulate the interfacial ion movement, and this kind of ion regulation effect can be applied to both anode and cathode sides. More importantly, a highly reversible Zn anode at a high current density of 160 mA cm^−2^ enables the fast-charge aqueous coin cell with noticeable stability (160 mA cm^−2^, 12,000 cycles) and fast-charge pouch cell with good cycling performance (56 mA cm^−2^, over 100 cycles) (Supplementary Fig. 24). Finally, we perform initial proof of concept studies to show that the findings are also relevant for achieving reversible, fast charge characteristics at reactive metal electrodes like Li tested in non-aqueous electrolyte environment.

## Methods

### Materials

ZnSO_4_·7H_2_O, ZnCl_2_, ZnBr_2_, ZnI_2_, Polyethylene glycol 300 (PEG300) and Deuterium oxide (D_2_O, ≥99.96%) were purchased from Sigma-Aldrich. In all, 0.25 mm Zn foil (99.994%) was bought from Alfa Aesar; 0.1 mm Zinc foil (99.999%), AC, DOL, LiNO_3_, LiTFSI and LiI were bought from Sigma. Chemicals are used as-is without any modifications. For 20-μm Zinc foil, 0.1 mm Zinc foil was rolled many times until reaching the desired thickness. Deionized water was obtained from Milli-Q water purification system. The resistivity of the deionized water is 18.2 MΩ cm^−1^ at room temperature. ZnSO_4_·7H_2_O, ZnCl_2_, ZnBr_2_ and ZnI_2_ were dissolved in deionized water to prepare 0.05 M, 0.5 M, 1 M electrolyte, respectively. Free-standing interwoven carbon fibers (Plain carbon cloth 1071 HCB) were purchased from Fuel Cell Store.

### Preparation of electrolytes

For electrodeposition and cell performance characterization, ZnSO_4_·7H_2_O, ZnCl_2_, ZnBr_2_ and ZnI_2_ were dissolved in deionized water to prepare 1 M electrolyte, respectively. 5 wt.% PEG300 was added to the 1 M ZnCl_2_, ZnBr_2_ and ZnI_2_ electrolyte, respectively to created ZnX_2_ (X = Cl, Br, I) polymer electrolyte.

For Li-containing non-aqueous electrolyte solution, 2 M LiTFSI, 0.5 M LiNO_3_ and 1 M LiI were added to the DOL solvent. For polymer-containing electrolyte, 1 wt% PEG400 was added.

### Electrochemical measurements

Galvanostatic charge/discharge performance of coin cells were tested on Neware battery test systems at room temperature (25 ± 1 °C). No climatic/environmental chamber was used. Electrochemical studies were performed using CR2032 coin cells. The area of electrodes in this study is 0.3175 cm^2^. Half cells were assembled with 0.25 mm Zn foil and carbon cloth and separated by a glass fiber (GF/A, WHA1821047, thickness 0.68 mm, pore size 1 μm). In each coil cell, ~100 μL electrolyte was added by pipette.

In the Zn||I_2_-AC coin cell, the electrolyte was 1 M ZnI_2_ with/without 5 wt.% PEG300. The cathode is prepared by mixing AC, Super P carbon and polyvinylidene fluoride at a weight ratio 8:1:1. The mass loading of AC is 5 mg cm^−2^. In the Zn||MnO_2_ coin cell, α-MnO_2_ was synthesized via a conventional hydrothermal route, in which a solution that contains 5 mM KMnO_4_ and 0.3 M HCl was kept at 140 °C for 18 h. All coin cells were assembled at the air.

For pouch cell, 20-μm zinc foil was assembled with cathode materials and separated by a Celgard 3501 membrane. Single-layer electrode was used in pouch cell. The area of the electrode is 9 cm^2^. The pouch cells were assembled in Ar-filled glovebox. The specific energy of pouch cell was calculated by:

$${{{{{{\rm{Specific}}}}}}}\,{{{{{{\rm{energy}}}}}}}\,\left({{{{{{\rm{Wh}}}}}}}\,{{{{{{{\rm{kg}}}}}}}}^{{{{{{\rm{-}}}}}}1}\right)=\frac{{{{{{{\rm{Average}}}}}}}\,{{{{{{\rm{discharge}}}}}}}\,{{{{{{\rm{voltage}}}}}}}\ \left(V\right)\times {{{{{{\rm{discharge}}}}}}}\,{{{{{{\rm{capacity}}}}}}}({Ah})}{{{{{{{\rm{Total}}}}}}}\,{{{{{{\rm{mass}}}}}}}\,({{{{{{\rm{kg}}}}}}},\,{{{{{{\rm{active}}}}}}}\,{{{{{{\rm{materials}}}}}}}\,{{{{{{\rm{on}}}}}}}\,{{{{{{\rm{cathode}}}}}}}\,{{{{{{\rm{side}}}}}}}+{{{{{{\rm{Zn}}}}}}}\,{{{{{{\rm{anode}}}}}}})}$$

For Li||I_2_-AC cell, all procedures are the same as Zn||I_2_-AC cell. The electrolyte is DOL + 2 M LiTFSI + 2 M LiI + 0.5 M LiNO_3_ + 1% PEG400 and the membrane is Celgard 2400. The cathode side is AC. The Li||I_2_-AC cell was assembled in Ar-filled glovebox.

EIS measurements was performed using a Solartron in a frequency range from 50 to 1 HZ with an AC polarization of 10 mV. The measurements used two-electrode zinc symmetric cells. Overall, 56 points were collected for each test. Before carrying out the EIS measurement, the open-circuit voltage–time applied was 2 h.

For i–v curve, a three-electrode system, including a working electrode made of glassy carbon, a counter electrode made of zinc foils and a reference electrode (AgCl/Ag), was used to measure the i–v curve of these four zinc salts electrolyte with various concentration (0.05 M, 0.5 M and 1 M). The glassy carbon electrode was purchased from Pine Research. The scan rate of all experiments is 5 mV s^−1^. Limiting current can be extracted from the i–v curve. During the scanning process, no bubbling is observable near the working electrode, which is attributable to the sluggish kinetics of the H_2_ evolution reaction in this system.

Exchange current: cyclic voltammetry was performed using a CHI 600E electrochemical workstation and symmetrical zinc cells, in which the glass fiber is used as a separator. Tafel plots of 1 M ZnSO_4_ and various ZnX_2_ aqueous electrolytes derived from cyclic voltammetry measurements with a scan rate of 0.2 V s^−1^. Exchange current density calculated from the Tafel plots.

### Characterization of materials

For ^1^H NMR sample preparation, 25 µL PEG300 was first diluted in 500 µL D_2_O, and then desired amount of ZnSO_4_·7H_2_O, ZnCl_2_, ZnBr_2_ and ZnI_2_ were slowly dissolved in this solution. Various amount of ZnSO_4_·7H_2_O, ZnCl_2_, ZnBr_2_ and ZnI_2_ were added in 500 µL D_2_O with 25 µL PEG300 solution to prepared 0.1 M, 0.5 M, 1 M, 1.5 M, 2 M and 4 M ZnI_2_ polymer electrolytes.

For Ellipsometer experiments, a silicon wafer with a 2 × 2 cm^2^ area was used. For each silicon wafer, we tested three points and get the average thickness. For each kind of electrolyte, we tested three samples (three silicon wafers), which means totally we tested nine times for the PEG adsorption thickness and then extracted the average value.

Field-emission scanning electron microscopy was carried out on Zeiss Gemini 500 Scanning Electron Microscope equipped with Bruker energy dispersive spectroscopy detector to study the electrodeposition morphology of Zn. NMR ^1^H spectra were collected on a Bruker AVIIIHD (H, 500 MHz) spectrometer with a broad band Prodigy cryoprobe and referenced to D_2_O solvent shifts (D_2_O = 4.78 ppm). Linear sweep voltammetry was performed using a CH 600E electrochemical workstation. Impedance measurements of all electrolytes were measured using frequency–domain dielectric relaxation measurements in the range 10^7^–10^−1^ Hz using a Novocontrol Broadband dielectric spectrometer. ATR-FTIR spectra were conducted using a Thermo Scientific spectrometer. Renishaw inVia confocal Raman microscope is used for Raman tests of electrolytes (excitation wave-length: 785 nm).

QCM-D measurements were performed with Q-sense Explorer system (Biolin Scientific AB) at 25 °C. Fundamental frequency and three harmonics (third, fifth and seventh) were recorded simultaneously along with the corresponding dissipation factors. Gold coated AT-cut quartz crystal (fundamental frequency ~4.95 MHz) was purchased from Q-sense (QX301). Before the experiment, the crystal was cleaned by immersing in 5:1:1 solution of water, ammonia (25%) and hydrogen peroxide (30%) for 5 min at 75 °C, followed by thorough rinsing with deionized water. The crystal was subsequently dried with nitrogen gas and finally UV treated for 10 min in UV/Ozone Procleaner^TM^ (Bioforce, Nanosciences). 1 M ZnSO_4_ and Zn halide solution was used as the buffer solution and a stable baseline was obtained before switching to 1 M ZnSO_4_ and Zn halide solution + 5 wt% PEG300 solution. The data obtained from QCM-D was modeled using the Q-tools software (Q-sense). “Voigt” viscoelastic model was used to model third, fifth and seventh harmonics. This model assumes a homogeneous adsorbed layer. Fluid density and viscosity were taken to be 1638 kg m^−3^ and 0.001 kg ms^−1^, respectively. The density of the adsorbed layer was assumed to be 1125 kg m^−3^ (which is the density of the PEG300) for calculating viscoelastic properties. Descending incremental fitting was used. Direct measurement of ion-oligomer complex adsorption thickness was performed at ALPHA-SE ELLIPSOMETER. The data were collected through three angles (65°, 70°, 75°) and ten different adsorption sites of the silicon wafer, and then get the average adsorption thickness of different PEG300+ Zn salts electrolytes.

#### First-principles calculations

The bond formation of PEG-Zn-halide complexes was studied by ab initio quantum chemistry calculations using the General Atomic and Molecular Electronic Structure System (GAMESS-US), version 2019 R1 patch 1. All calculations were based on the restricted Hartree–Fork method, with a non-SMD solvation model of water (ε = 78.3553) and a gradient convergence tolerance of 0.0001 Hartree Bohr^–1^. An example of our GAMESS header can be found below. The initial geometries of the ZnX_n_ (H_2_ O)_m−n_ complexes were individually constructed in Avogadro ver1.2.0 and then optimized in GAMESS using a 6-31G** basis set assuming the atomic radius of Zn is 1.39 Å. Together with a linear PEG molecule and five free water molecules, each ZnX_n_ (H_2_ O)_m−n_ complex structure was further optimized in GAMESS using the same parameter settings. The degree of polymerization of PEG was chosen to be 5-unit to resemble the molecular weight used in the experiment (~300 g mol^−1^). After convergence, the bond lengths and bond angles were obtained from the equilibrium structure. HOMO and LUMO energy extracted from the ab initio model.

GAMESS header example:

$SYSTEM MWORDS = 125 $END

$PCMCAV RIN(1) = 1.39 $END

$PCM SOLVNT = WATER $END

$BASIS GBASIS = N31 NGAUSS = 6 NDFUNC = 1 NPFUNC = 1 $END

$CONTRL SCFTYP = RHF RUNTYP = OPTIMIZE ICHARG = −2 MAXIT = 100 $END

$STATPT OPTTOL = 0.0001 NSTEP = 1000 $END

$SCF DIRSCF = .T. $END

## Supplementary information

Supplementary Information

## Data Availability

All datasets generated and analyzed during the current study are available from the corresponding author upon reasonable request.
