# Supplementary material for: Production of fast-charge Zn-based aqueous batteries via interfacial adsorption of ion-oligomer complexes
Source: Nat Commun. 2022 Apr 27;13:2283. doi: 10.1038/s41467-022-29954-6 (PMC9046403; doi:10.1038/s41467-022-29954-6)
Supplement: Supplementary file 1 — Supplementary Information [file 41467_2022_29954_MOESM1_ESM.pdf]

Supplementary Information

Production of fast-charge Zn-based aqueous batteries via interfacial adsorption of ion-oligomer complexes

Shuo Jin[1], Jiefu Yin[1], Xiaosi Gao[1], Arpita Sharma[1], Pengyu Chen[1], Shifeng Hong[2], Qing Zhao[1], Jingxu zheng[2], Yue Deng[2], Yong Lak Joo[1], Lynden A. Archer[1*]

1. Robert Frederick Smith School of Chemical and Biomolecular Engineering, Cornell University, Ithaca, NY, 14853, USA

2. Department of Materials Science and Engineering, Cornell University, Ithaca, NY, 14853, USA.

** Corresponding author: laa25@cornell.edu*

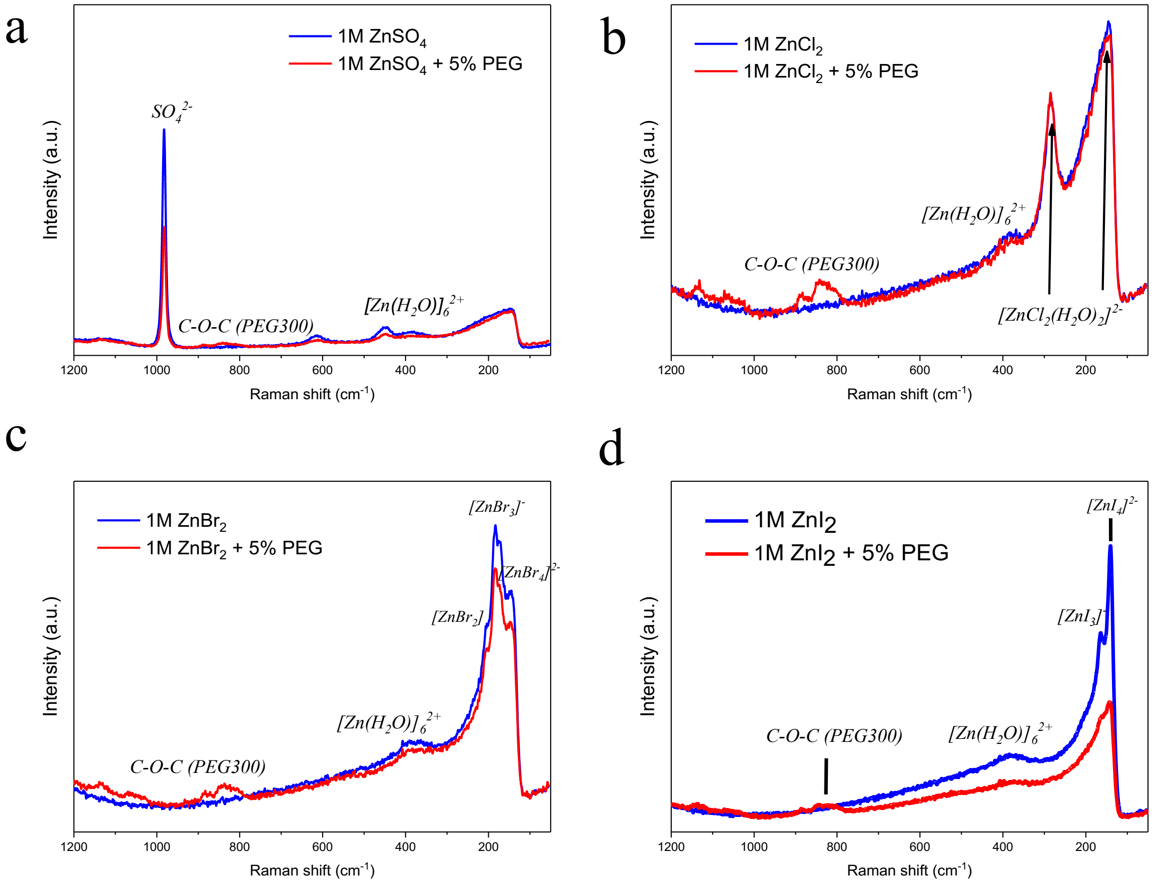

Supplementary Figure 1. Raman spectrum of (a) 1M $ZnSO_4$ and 1M $ZnSO_4$ + 5% PEG electrolytes, (b) 1M $ZnCl_2$ and 1M $ZnCl_2$ + 5% PEG electrolytes, (c) 1M $ZnCl_2$ and 1M $ZnCl_2$ + 5% PEG electrolytes, (d) 1M $ZnCl_2$ and 1M $ZnCl_2$ + 5% PEG electrolytes.

Note: Here, although the previous report gave the structure of $[Zn(H_2O)_4Cl_2]^{2+}$, our *Ab initio* calculation shows that $[Zn(H_2O)_2Cl_2]^{2+}$ is more stable than $[Zn(H_2O)_4Cl_2]^{2+}$), especially after bonding with PEG. [1]

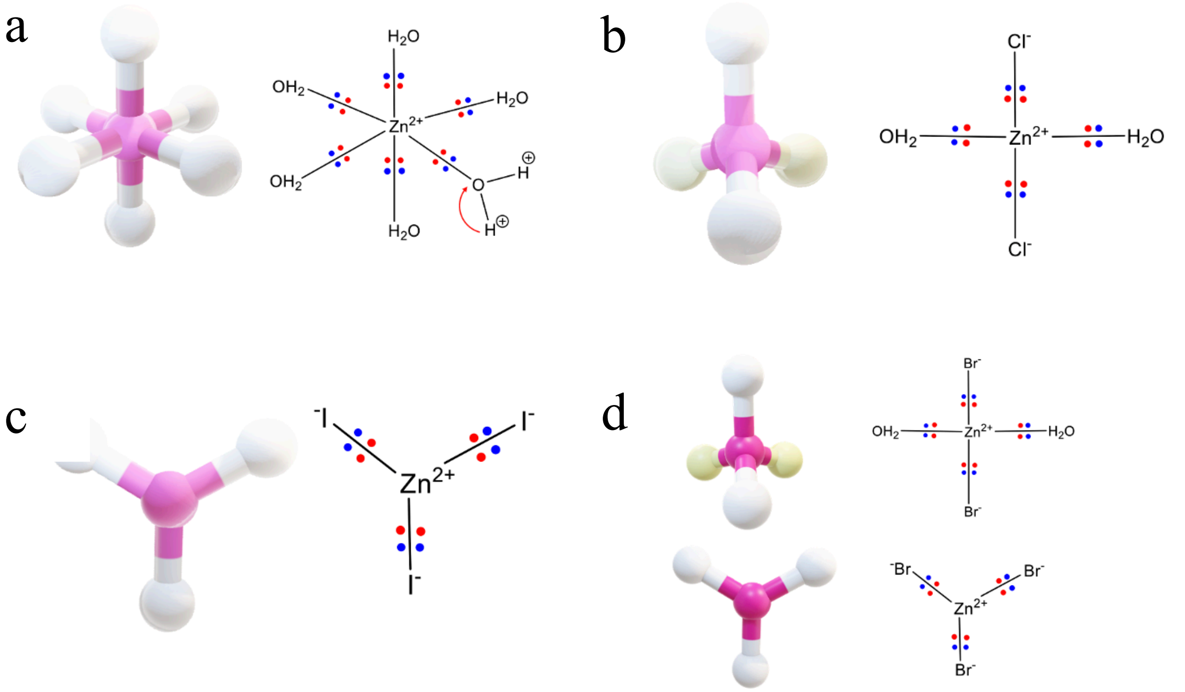

Supplementary Figure 2. Initial Zn^{2+} solvation ions in (a) $ZnSO_4$ electrolytes, $[Zn(H_2O)6]^{2+}$; (b) $ZnCl_2$ electrolytes, $[Zn(H_2O)_2Cl_2]^{2+}$ (Here, although previous report gave the structure of $[Zn(H_2O)_4Cl_2]^{2+}$, our *Ab initio* calculation shows that $[Zn(H_2O)_2Cl_2]^{2+}$ is more stable than $[Zn(H_2O)_4Cl_2]^{2+}$); (c) ZnI_2 electrolytes, $[ZnI_3]^-$ (Here, only plot the structure of $[ZnI_3]^-$, but also contains $[ZnI_4]^{2-}$,$[ZnI_2]$, $[ZnI]^+$). (d) $ZnBr_2$ electrolytes, $[Zn(H_2O)_2Br_2]^{2+}$ and $[ZnBr_3]^-$.

a

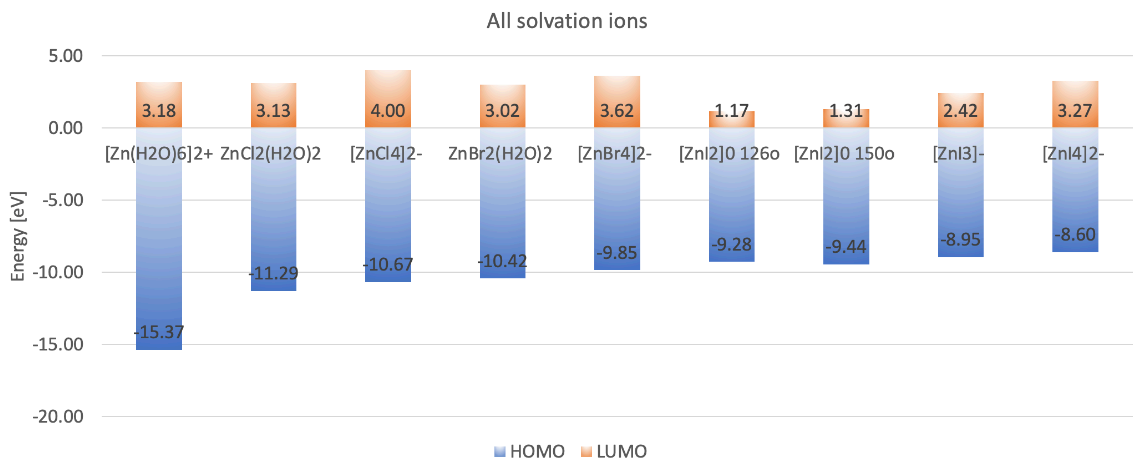

Supplementary Figure 3. HOMO and LUMO of different solvation ions in different electrolytes. The relatively low LUMO potential of unstable Zn-halide complex ($[ZnI_x]^{2-x}$) can explains the high reaction kinetics (high exchange current) in Zn halide electrolyte.

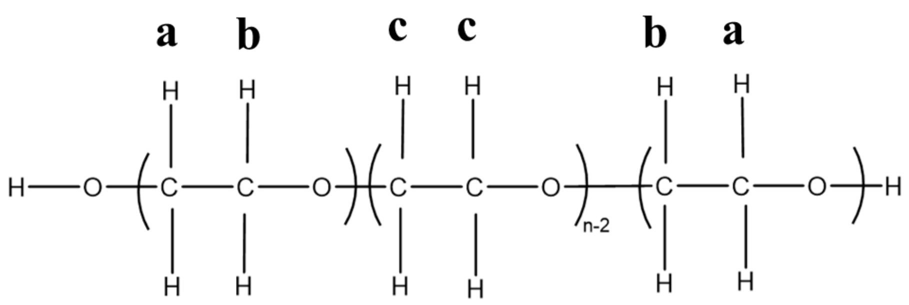

Supplementary Figure 4. Peak a, b and c represent different ^{1}H peaks of PEG300 backbone, showing at Figure 2.

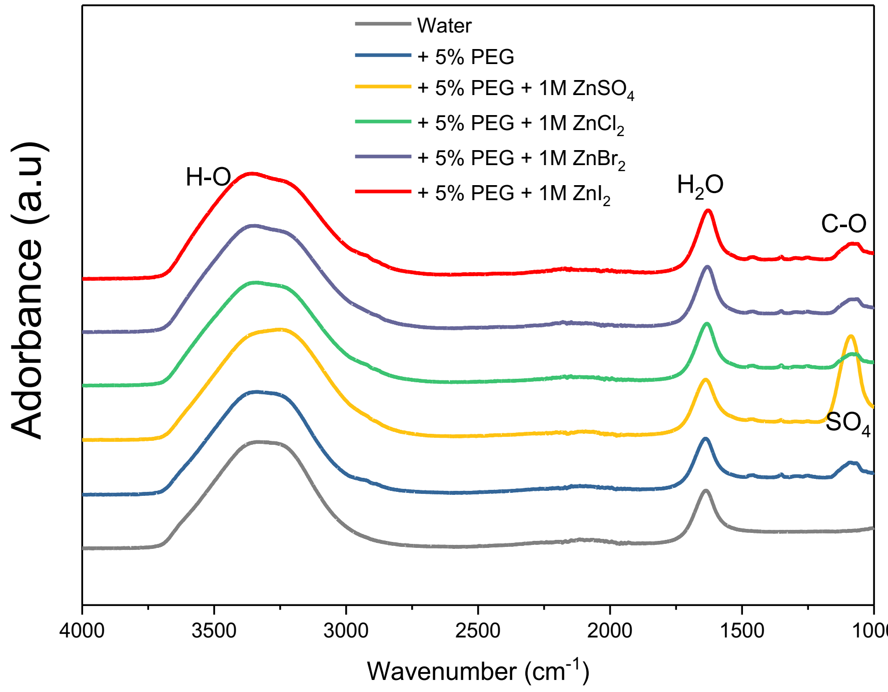

Supplementary Figure 5. Full Fourier-transform infrared spectroscopy (FT-IR) of 1M $ZnSO_4$ and various ZnX_2 (X=Cl, Br, I) aqueous electrolytes containing 5 wt% PEG300.

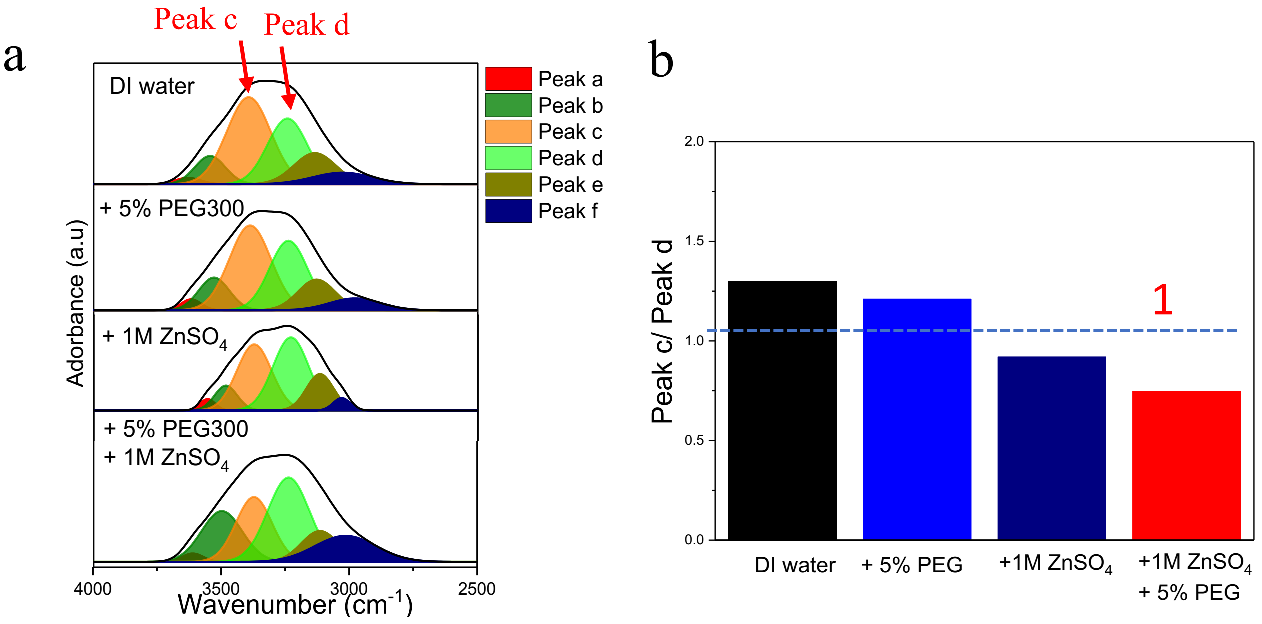

Supplementary Figure 6. FT-IR of hydrogen bond in (a) DI water, DI water + 5wt% PEG300, DI water +1M $ZnSO_4$, and DI water + 5wt% PEG300+1M $ZnSO_4$. (b) The ratio between PEAK c/PEAK d. PEAK c represents weak hydrogen bonds, Peak d represents strong hydrogen bonds. The ratio represents the formation number of hydrogen bonds.

In Supplementary Figure 6, the peaks a-f can be assigned to the different levels of H-bond cooperativity: a, free; b, 0 and 1; c, 2; d, 3; e, 3'; f, 4. Larger values indicate higher hydrogen bond cooperativity. Combined with our NMR results, we can say that higher H-bond cooperativity means the interaction between solvation ions and PEG is much stronger. In FTIR, the area (ratio) of peak c and peak d accounts for over 70% and the change of other peaks (a, b, e, f) is minor. Hence, the ratio of peak c/peak d can reflect the degree of hydrogen bond cooperativity.

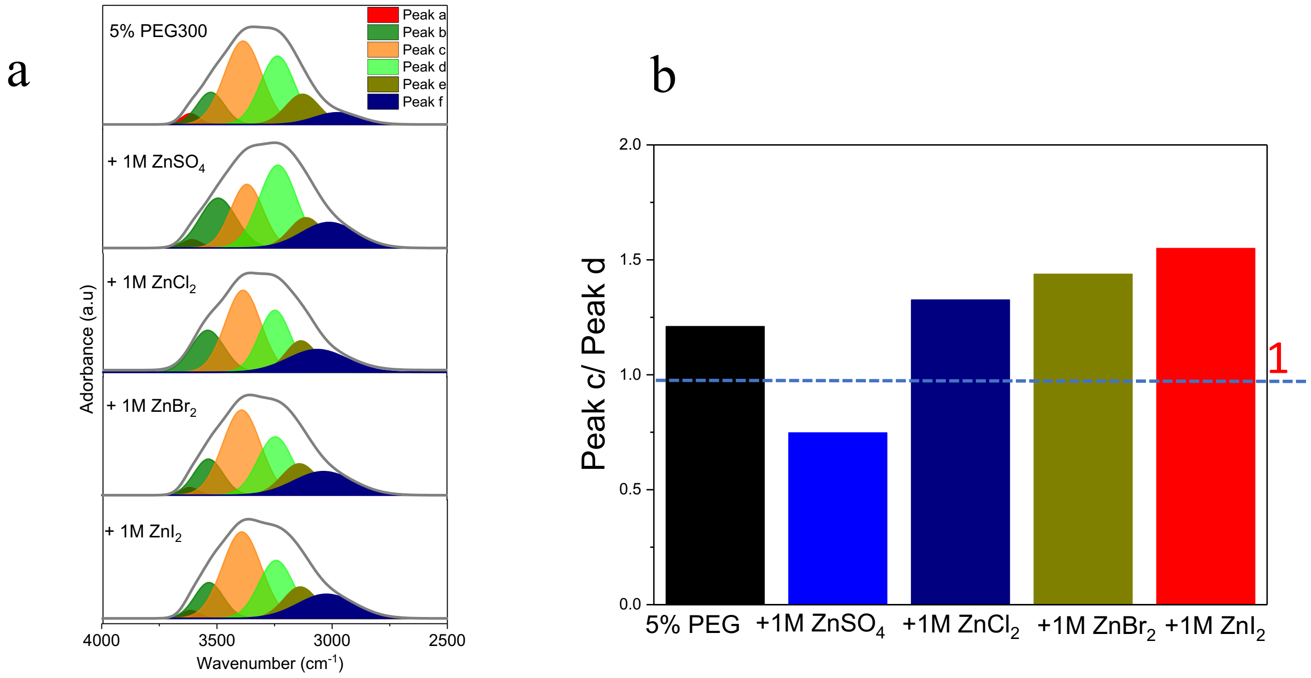

Supplementary Figure 7. FT-IR of (a) 5wt% PEG300 solution, and 1M $ZnSO_4$ and various ZnX_2 (X=Cl, Br, I) aqueous electrolytes containing 5 wt% PEG300. (b) The ratio between PEAK c/PEAK d.

In Supplementary Figure 7, the peaks a-f can be assigned to the different levels of H-bond cooperativity: a, free; b, 0 and 1; c, 2; d, 3; e, 3'; f, 4. Larger values indicate higher hydrogen bond cooperativity. Combined with our NMR results, we can say that higher H-bond cooperativity means the interaction between solvation ions and PEG is much stronger. In FTIR, the area (ratio) of peak c and peak d accounts for over 70% and the change of other peaks (a, b, e, f) is minor. Hence, the ratio of peak c/peak d can reflect the degree of hydrogen bond cooperativity. For $ZnCl_2$ + 5% PEG electrolytes, the hydrogen bond cooperativity is weaker than $ZnSO_4$ + 5% PEG electrolytes due to the different solvation ion structures ($[ZnCl_2(H2O)_2]$, $[Zn(H_2O)_6]^{2+}$), which leads to the different peak c/peak d ratio and different NMR shield results. Here, $[Zn(H_2O)_6]^{2+}$ can bond with more free water molecules compared with $[ZnCl_2(H_2O)_2]$.

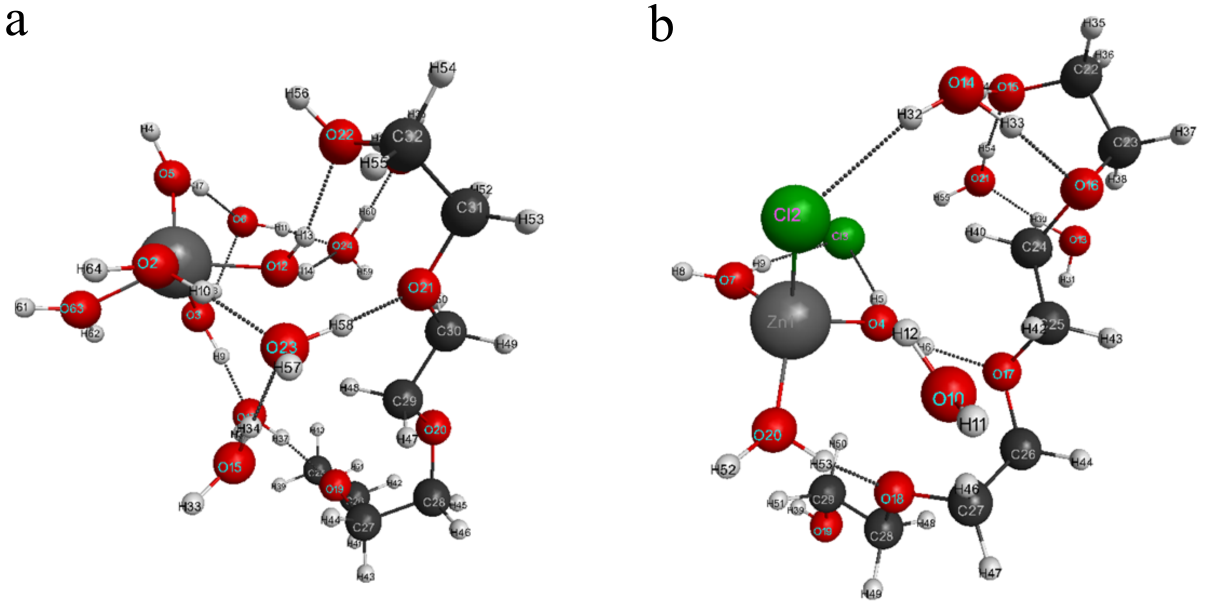

Supplementary Figure 8. ***Ab initio*** **calculation molecular structures of** (a)$[Zn(H_2O)_6]^{2+}$, water molecules and PEG300 in $ZnSO_4$ electrolytes; (b) $[Zn(H_2O)_2Cl_2]^{2+}$, water molecules and PEG300 in $ZnCl_2$ electrolytes.[2,3] The bond parameters are reported in **Supplementary Table 1 and Supplementary Table 2.**

a

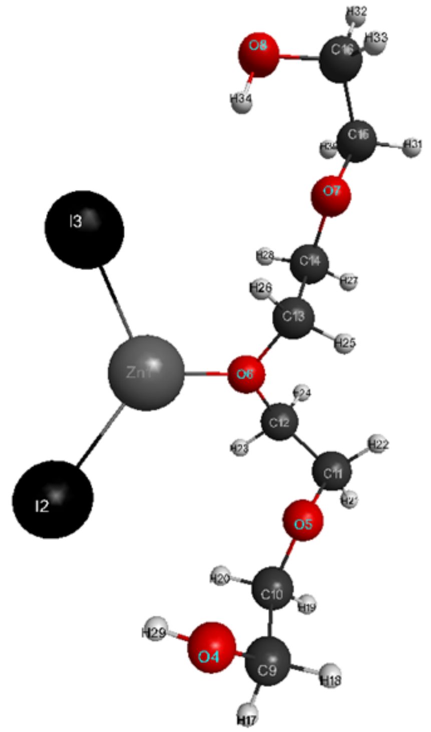

b

Frame 1 (initial geometry)	Frame 5	Frame 10	Frame 50	Frame 113 (equilibrium)
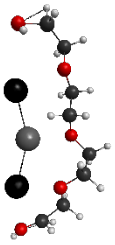	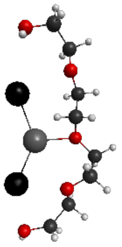	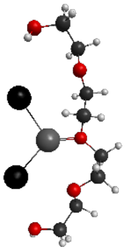	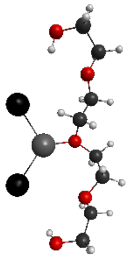	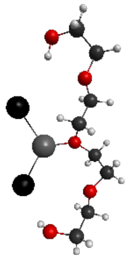

Supplementary Figure 9. *Ab initio* calculation molecular structures of [ZnI_2] and PEG300 in ZnI_2 electrolytes. (a) equilibrium states; (b) equilibrium process between [ZnI_2] and PEG300.[2,3] The bond parameters are reported in **Supplementary Table 3.**

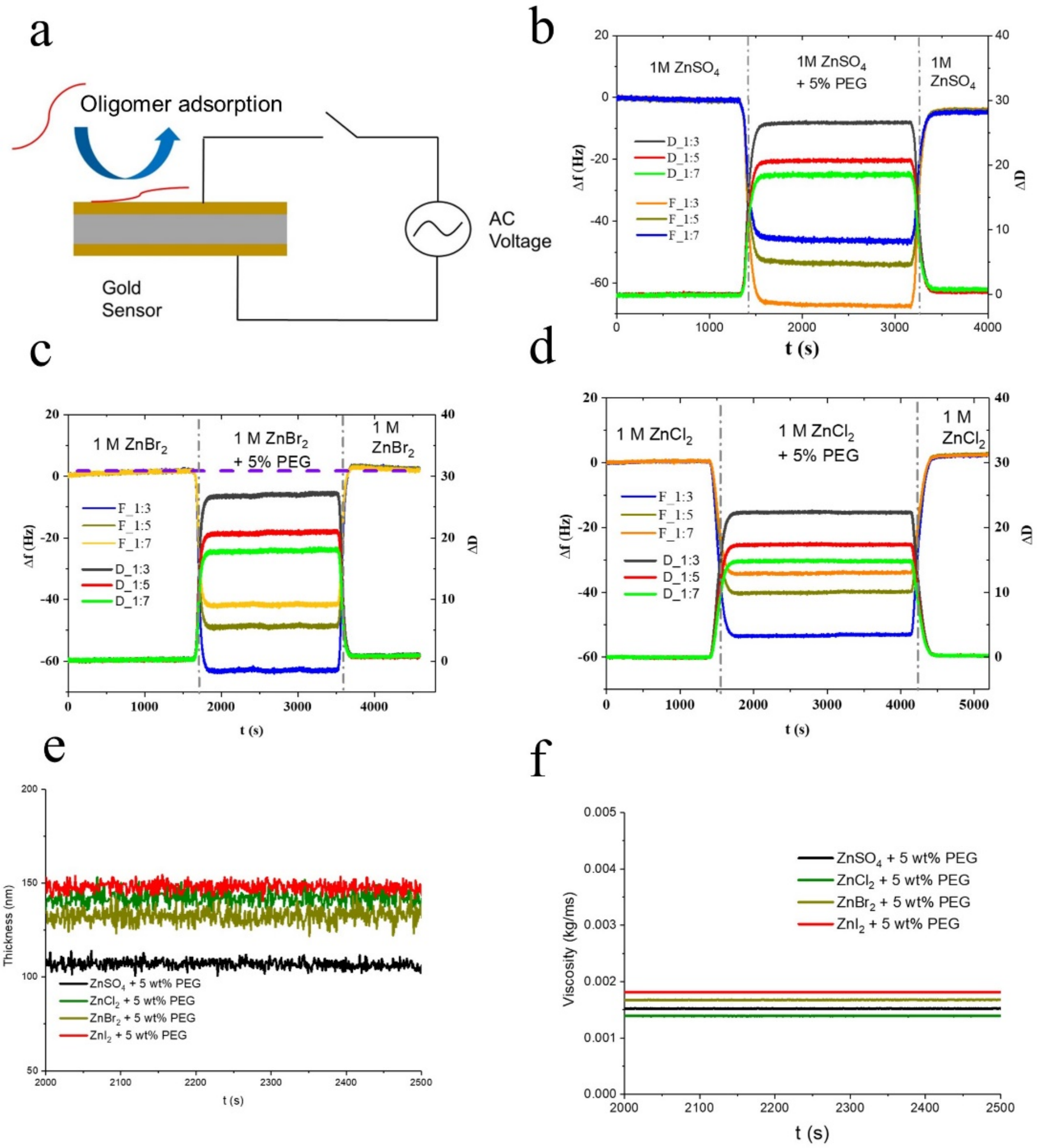

Supplementary Figure 10. Quartz Crystal Microbalance (QCM) measurements and analysis of (a)Scheme illustrates the QCM equipment. (b) 1M $ZnSO_4$ and 1M $ZnSO_4$ + 5% PEG electrolytes, (c) 1M $ZnCl_4$ and 1M $ZnCl_2$ + 5% PEG electrolytes, (d) 1M $ZnBr_2$ and 1M $ZnBr_2$ + 5% PEG electrolytes. Ion-oligomer complex adsorption thickness (e) and interfacial viscosity (f) extracted from (b) (c) and (d).

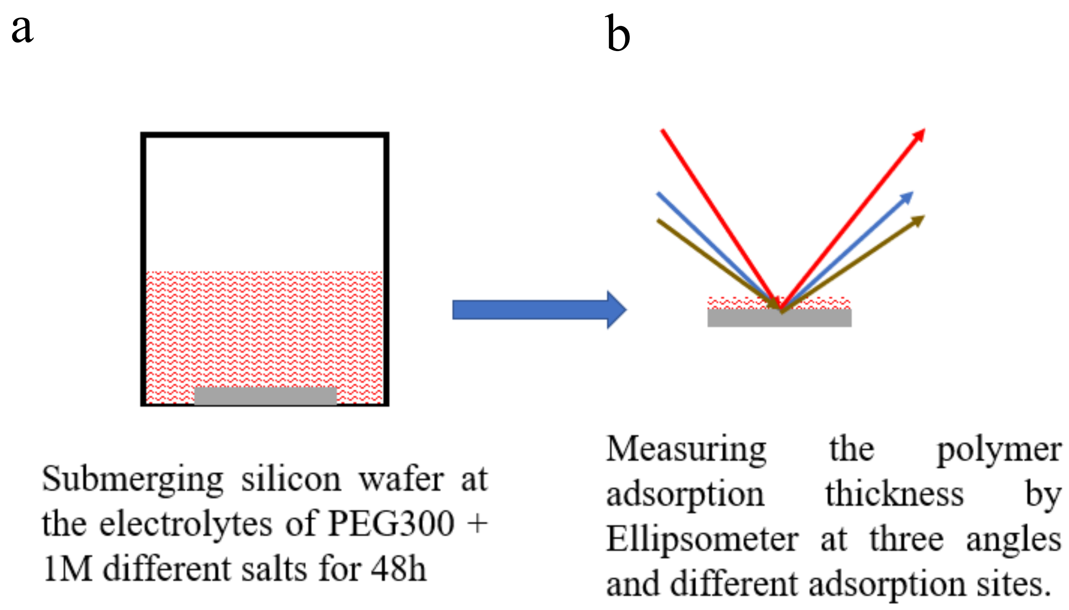

Supplementary Figure 11. Alpha-SE Ellipsometry analysis of ion-oligomer complex adsorption thickness (a) submerging silicon wafer at the electrolytes of PEG300 + 1M different salts for 48h, the read lines represent PEG solution. (b) Measuring the ion-oligomer complex adsorption thickness by ellipsometer at three different angles.

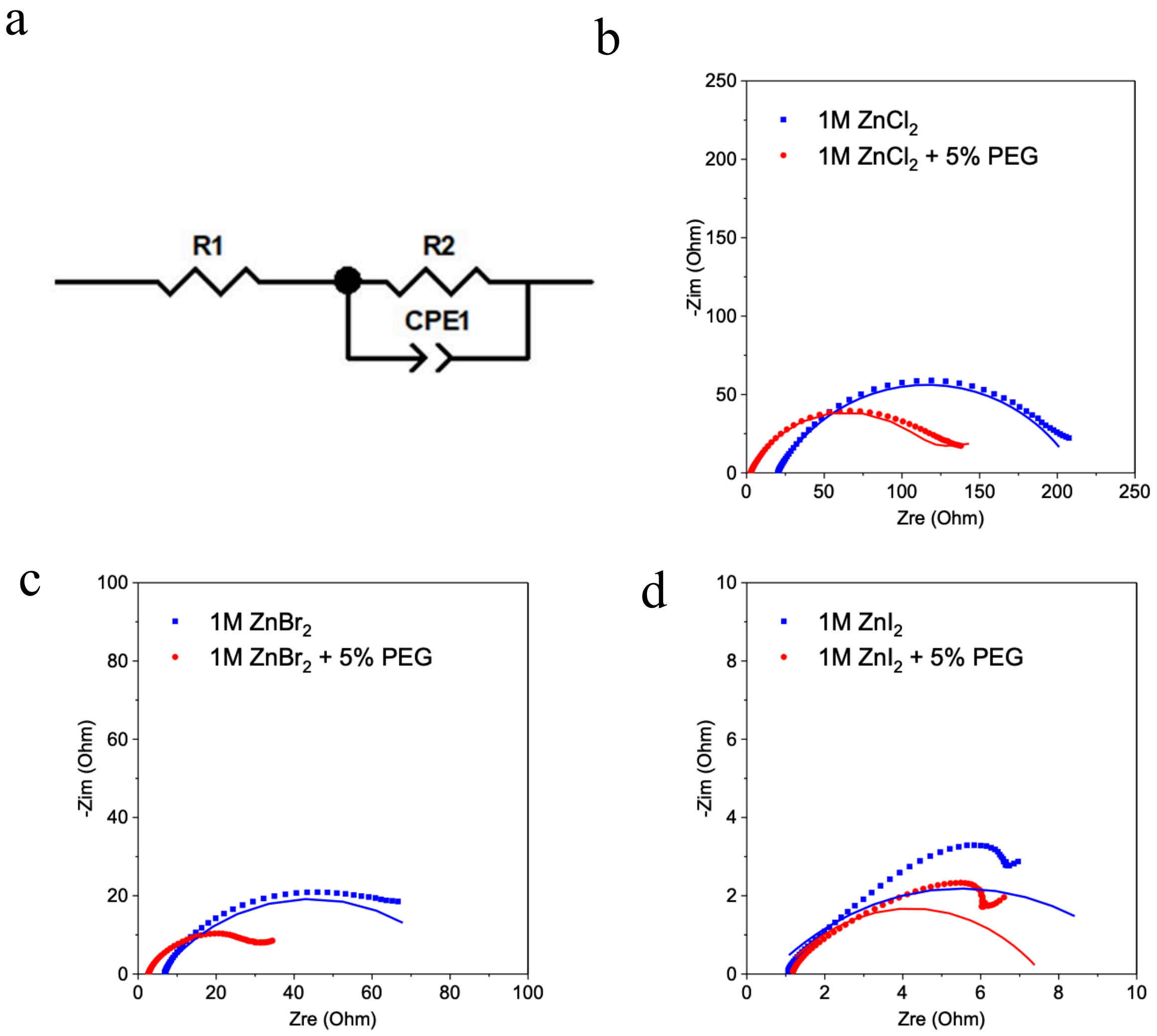

Supplementary Figure 12. (a) Equivalent circuit model for all raw data fitting process. EIS analysis of (b) 1M $ZnCl_2$ with/without (5 wt%) PEG300; (c) 1M $ZnBr_2$ with/without (5 wt%) PEG300; (d) 1M ZnI_2 with/without (5 wt%) PEG300. The measurements were all performed in Zn||Zn symmetric cells; the raw impedance values are reported as symbols in the plots. The lines through the data are best fit curves obtained using the equivalent circuit model depicted on (a).

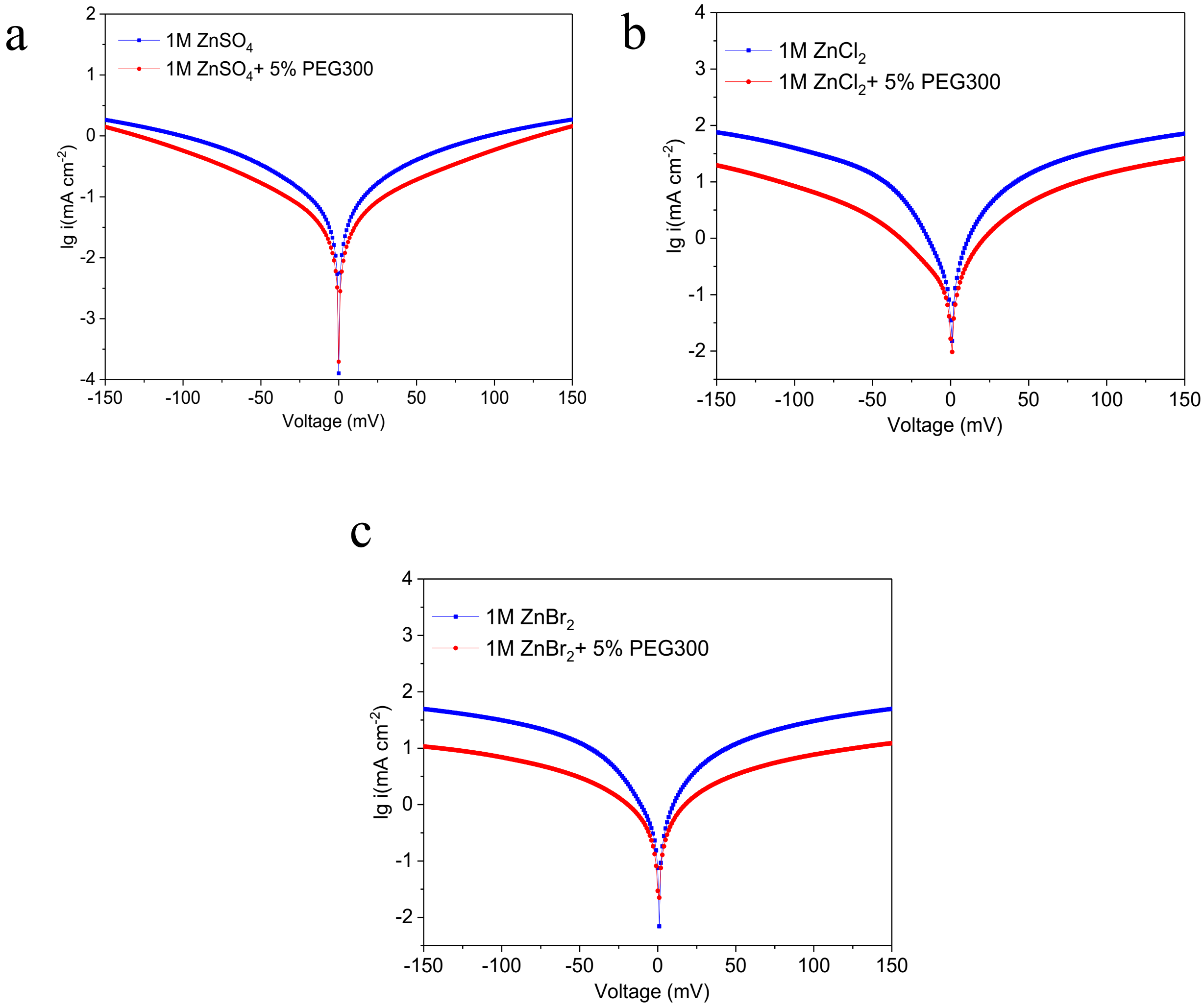

Supplementary Figure 13. Tafel plots of (a) 1M $ZnSO_4$ with/without (5 wt%) PEG300; (b) 1M $ZnCl_2$ with/without (5 wt%) PEG300; (c) 1M $ZnBr_2$ with/without (5 wt%) PEG300. Scan rate 0.5V s^{-1}.

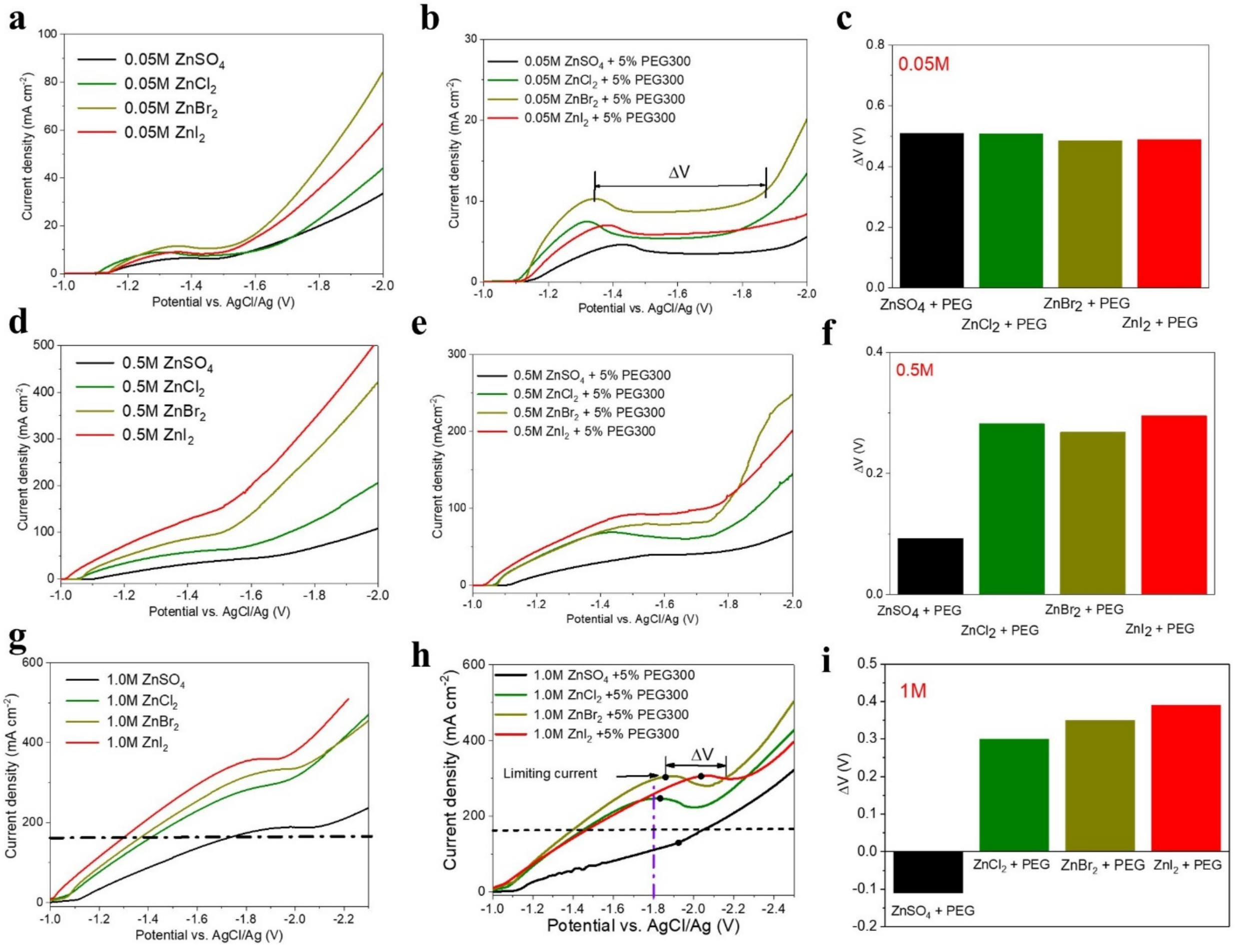

Supplementary Figure 14. The current-voltage (i-V) curve measured in a three-electrode cell. (a) 0.05M $ZnSO_4$ and various ZnX_2 aqueous electrolytes; (b) 0.05M $ZnSO_4$ and various ZnX_2 aqueous electrolytes; (d) 0.5M $ZnSO_4$ and various ZnX_2 aqueous electrolytes; (e) 0.5M $ZnSO_4$ and various ZnX_2 aqueous electrolytes containing 5 wt% PEG300. (g) 1M $ZnSO_4$ and various ZnX_2 aqueous electrolytes; (h) 1M $ZnSO_4$ and various ZnX_2 aqueous electrolytes containing 5 wt% PEG300; (c), (f), (i) voltage stability window ΔV extracted, respectively from, i-V data reported in (b), (e), and (h).

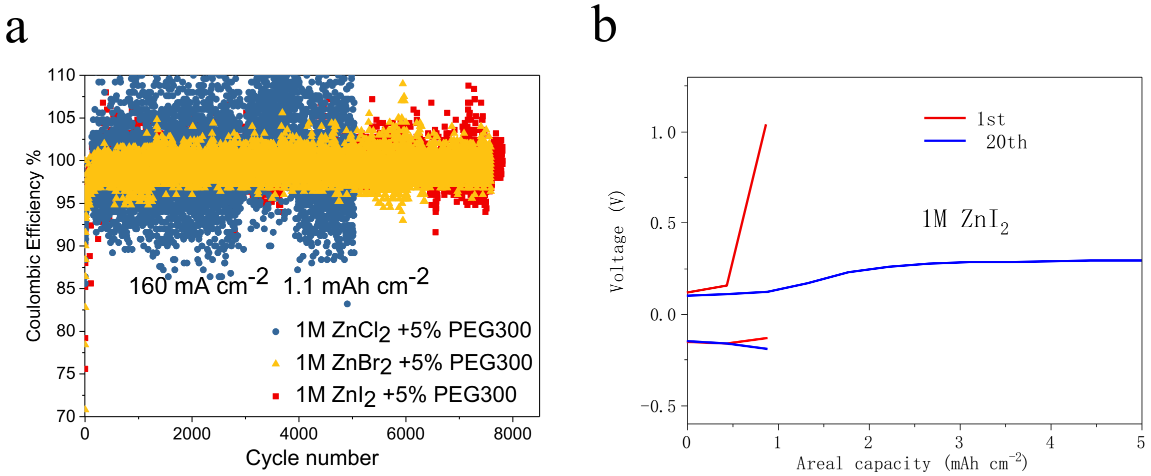

Supplementary Figure 15. Coulombic efficiency measured in Zn plate-strip experiments in Zn||carbon matrix half-cells in (a) 1M ZnX_2 (X=Cl, Br, I) +5 wt% PEG300 electrolytes at 160 mA cm^{-2}, 1.1 mAh cm^{-2}, (b) 1M ZnI_2 electrolyte.

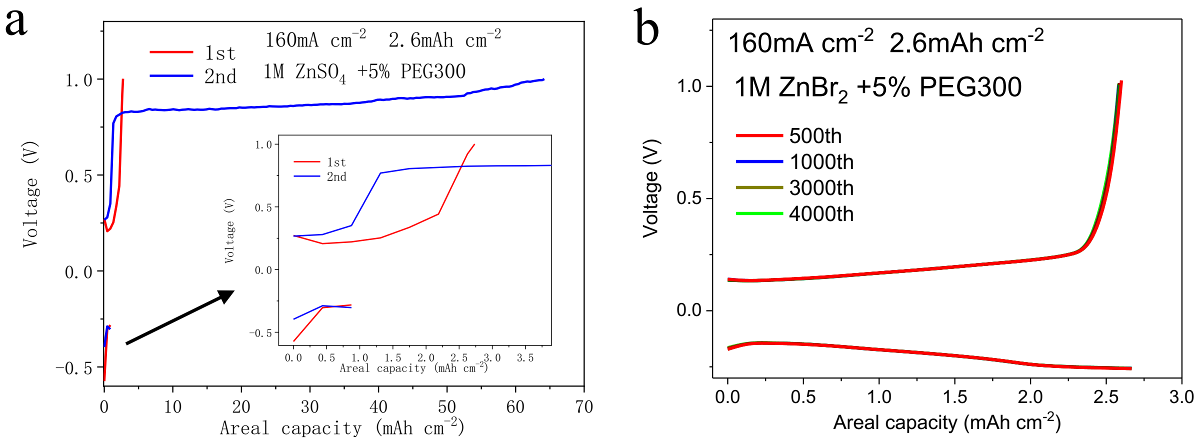

Supplementary Figure 16. Voltage-capacity curve for the corresponding Zn||carbon matrix half-cell in (a) $ZnSO_4$+5 wt% PEG300, inset is the amplification plot**;** (b) 1M $ZnBr_2$+5 wt% PEG300.

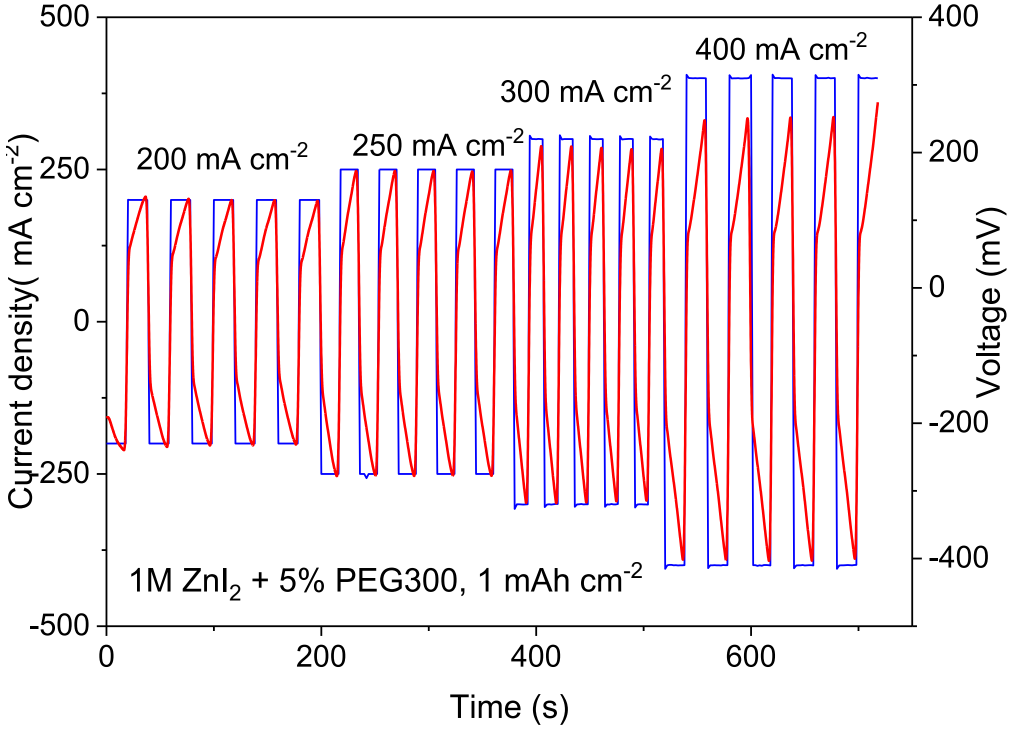

Supplementary Figure 17. Current-time and voltage-time curve for 1M ZnI_2 +5 wt% PEG300 at high current density. Zn symmetric cells were used here.

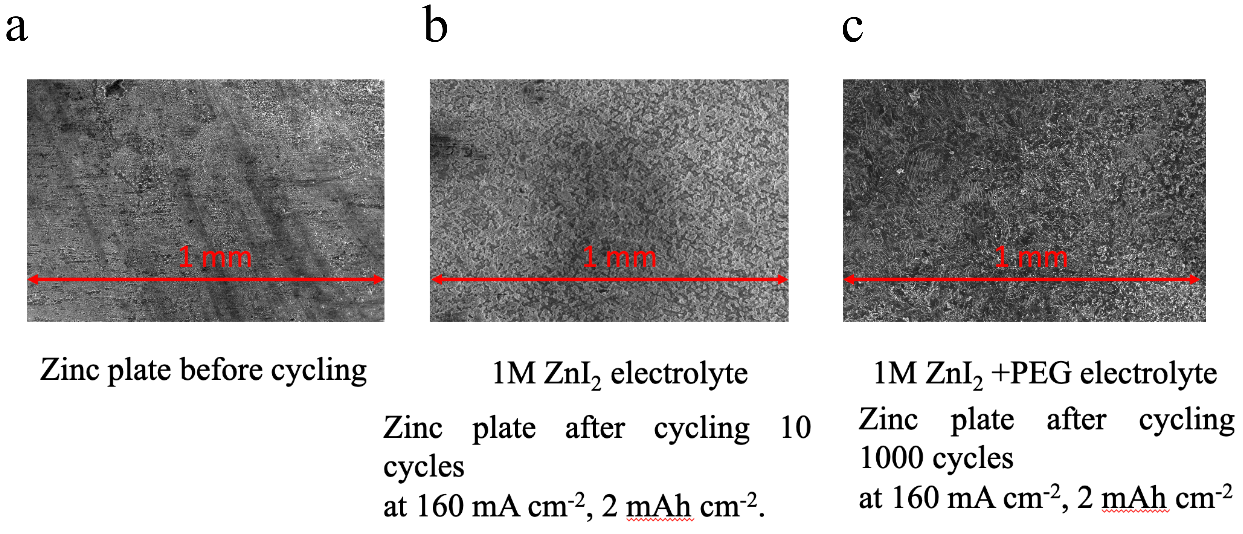

Supplementary Figure 18. The morphologies of Zn electrodeposits, before (a) and after cycling (b)(c). The scale is 1mm for all samples. For blank 1M ZnI_2 electrolyte solution, the symmetric cell was short-circuited after 10 cycles and then was disassembled. For 1M ZnI_2 + 5% PEG electrolyte solution, the symmetric cell was disassembled after 1000 cycles without short-circuiting.

The morphology of Zn electrodeposits formed in ZnI_2 + PEG electrolyte is observed to be smooth and uniform, even after 1000 cycles at a current density of 160 mA cm^{-2} and capacity of 2 mAh cm^{-2}. However, the electrodeposit morphology becomes noticeably coarser after 10 cycles in electrolytes that do not include the PEG oligomer additive.

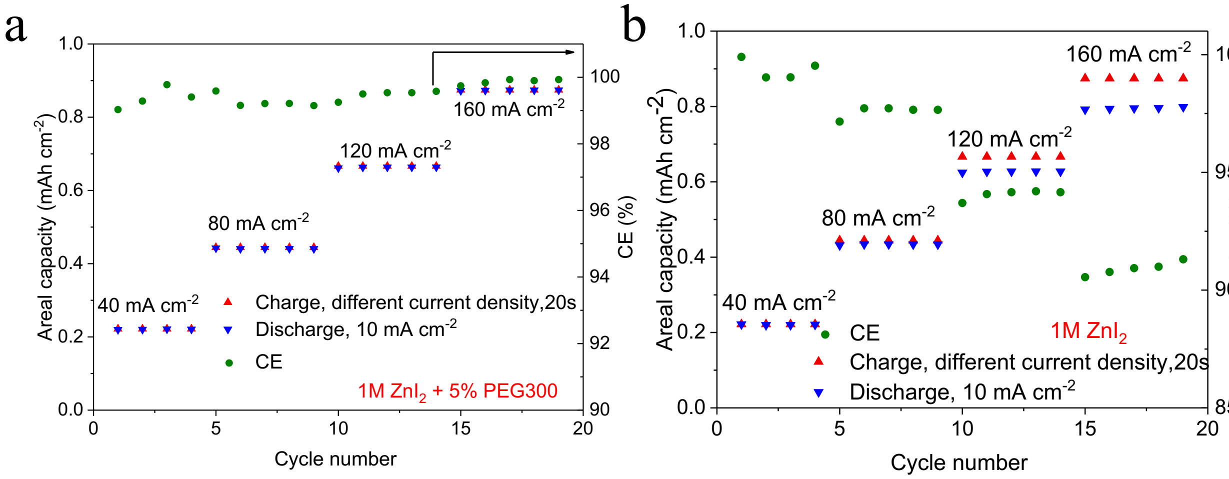

Supplementary Figure 19. Rate performance of Zn‖I_2-activeated carbon full cell measured in a 1M ZnI_2 electrolyte: (a) electrolyte containing 5 wt% PEG300; (b) same electrolyte as in a, but without the PEG 300 oligomer. The charging process utilized varying current densities of 40 mA cm^{-2}, 80 mA cm^{-2}, 120 mA cm^{-2}, 160 mA cm^{-2} for 20s, and the discharge process was performed at a fixed current density of 10 mA cm^{-2}.

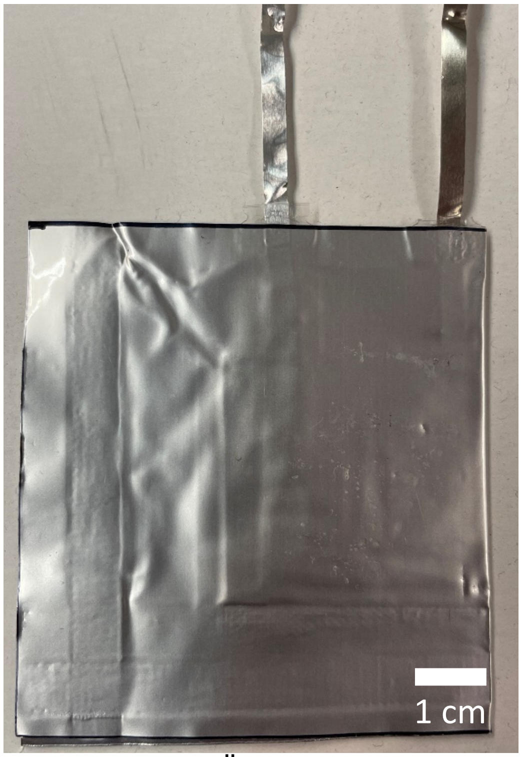

Supplementary Figure 20. Pouch cell of Zn‖I_2 (AC) full cell. The surface area of the electrodes is 9 cm^2. The areal capacity is 2.78 mAh cm^{-2}.

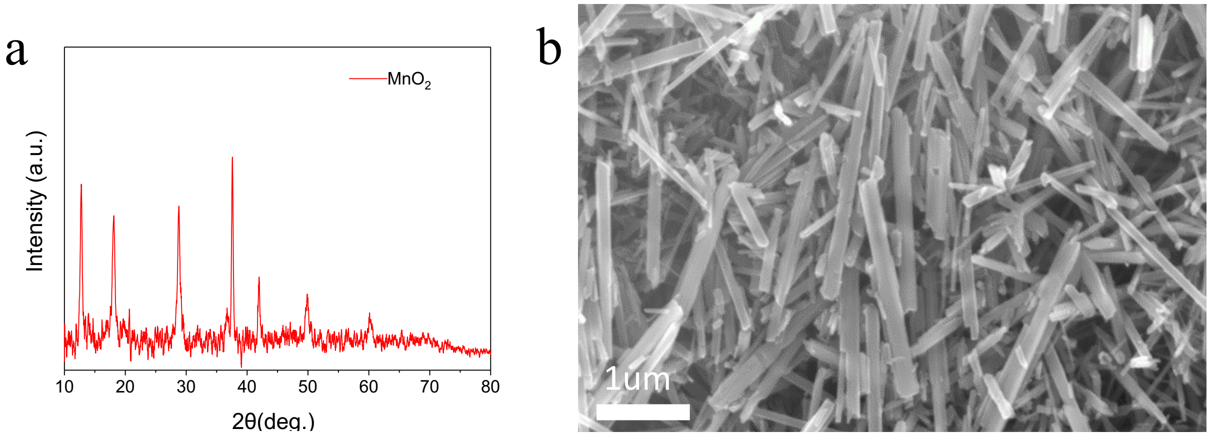

Supplementary Figure 21. (a) X-ray Diffraction and (b) Scanning electron microscopy analysis of the MnO_2 material used for the cell studies.[4]

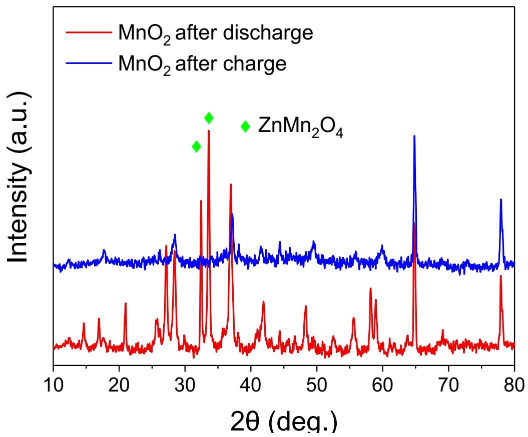

Supplementary Figure 22. The XRD of MnO_2 at 1M $ZnBr_2$ + 5% PEG electrolyte, which shows that Zn^{2+} is reversible with the regulation of adsorbed polymer layer. The cell was disassembled after one fully charge and discharge cycle and then the electrode was collected.

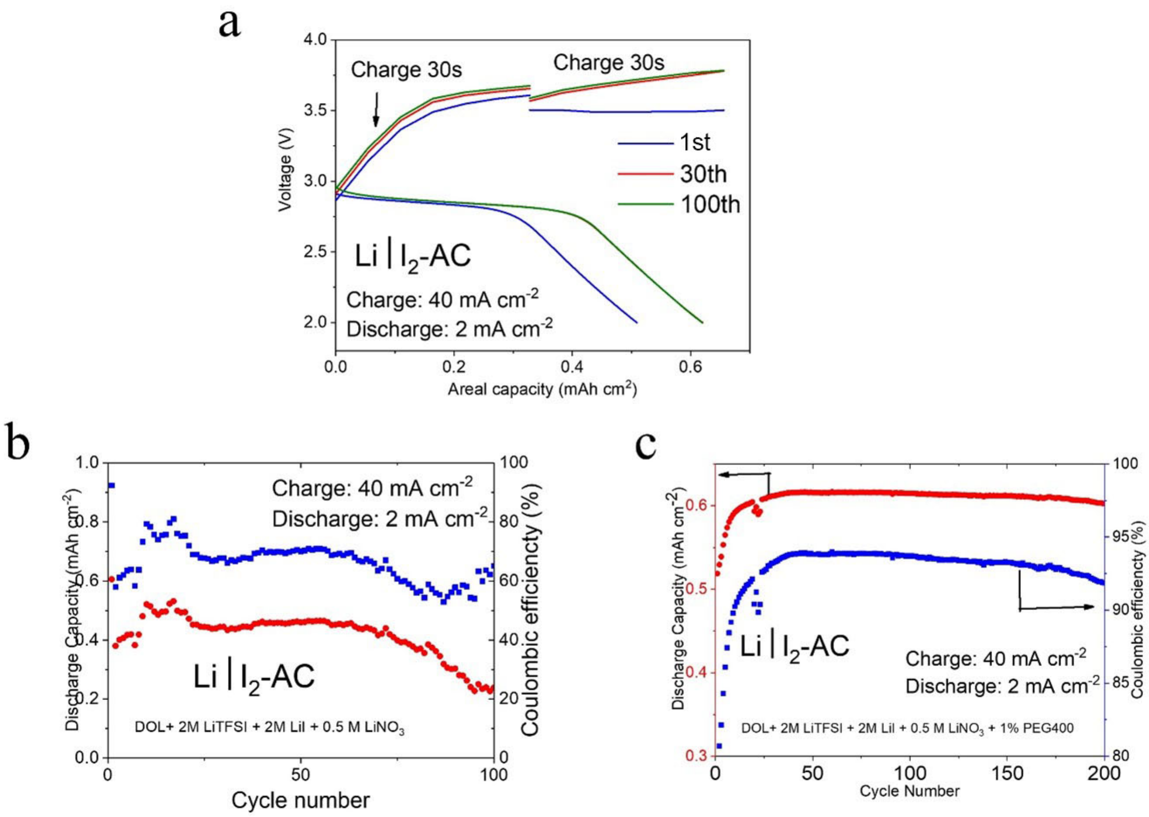

Supplementary Figure 23. Non-aqueous fast-charge Lithium-iodine cell. (a) The voltage-capacity curve of fast-charge Li||I_2 full cell in different cycles, charging at 40 mA cm^{-2} for 30s, resting for 10s, charging another 30s and then discharging at 2 mA cm^{-2}. Here, we used coin cells and the amount of electrolyte is 100ul. The electrolyte is DOL + 2M LiTFSI + 2M LiI + 0.5 M $LiNO_3$+1% PEG400. (b)Discharge capacity and coulombic efficiency of fast-charge lithium-iodine cell without PEG300 additive. The electrolyte is DOL + 2M LiTFSI + 2M LiI + 0.5 M $LiNO_3$. (c) Discharge capacity and coulombic efficiency of fast-charge Li||I_2 full cell. The electrolyte is DOL + 2M LiTFSI + 2M LiI + 0.5 M $LiNO_3$+1% PEG400.

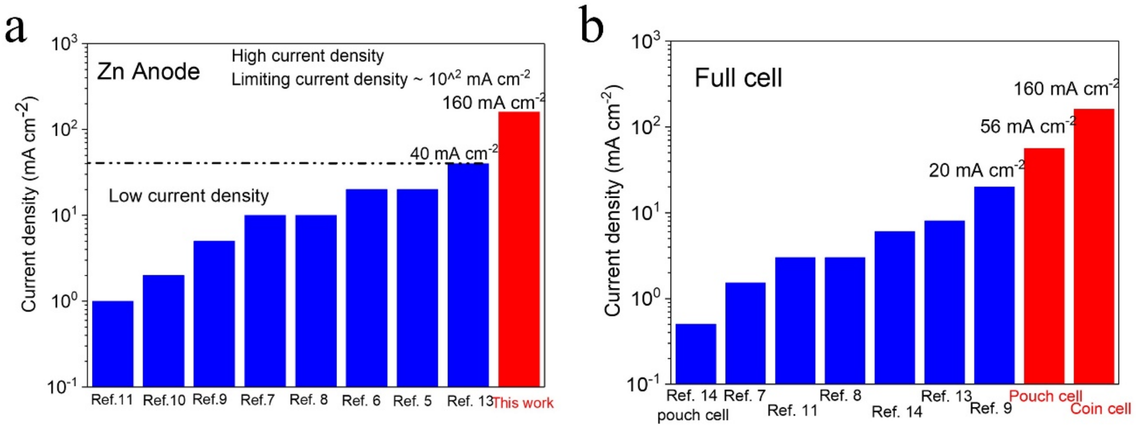

Supplementary Figure 24. Summary of the cell performance compared with other Zn anodes and aqueous Zn full cells.[5-14]

Supplementary Tables

$Zn(H_2O)_6$-$5H_2O$-5PEG

Hydrogen bonds in orange

Atom #	1		12		13		22
Element	Zn	-	O	-	H	...	(PEG end)O
Å		1.999		0.974		1.829	
order		0.323		0.666		0.088	

Atom #	1		2		10		23		58		21
Element	Zn	-	O	-	H	...	O	-	H	...	(PEG) O
Å		1.951		0.997		1.606		0.995		1.619	
order		0.380		0.583		0.157		0.606		0.142	

Atom #	1		3		9		17		37		19
Element	Zn	-	O	-	H	...	O	-	H	...	(PEG) O
Å		1.976		1.023		1.463		0.978		1.714	
order		0.370		0.528		0.186		0.674		0.116	

Supplementary Table 1. *Ab initio* calculation model of $[Zn(H_2O)_6]^{2+}$, water molecules and PEG300 in $ZnSO_4$ electrolytes. Tables give the bond length of the complex. Hydrogen bond length is labelled by orange.

$ZnCl_2(H_2O)_2$-$5H_2O$-5PEG

Atom #	1		2		32		14		33		16
Element	Zn	-	Cl	...	H	-	O	-	H	...	(PEG) O
Å		2.401		2.371		0.968		0.978		1.803	
order		0.375		<0.05		0.750		0.699		0.104	

Atom #	1		4		6		17
Element	Zn	-	O	-	H	...	(PEG) O
Å		1.893		1.005		1.552	
order		0.412		0.553		0.163	

Atom #	1		20		53		18
Element	Zn	-	O	-	H	...	(PEG) O
Å		1.882		1.013		1.503	
order		0.444		0.533		0.167	

Supplementary Table 2. ***Ab initio*** **calculation model of** $[Zn(H_2O)_2Cl_2]^{2+}$, water molecules and PEG300 in $ZnCl_2$ electrolytes. Tables give the bond length of the complex. Hydrogen bond length is labelled by orange.

ZnI_2 -5PEG

		Initial Å	Equilibrium Å	Equilibrium bond order
Zn1	I2	2.680	2.706	0.650
	I3	2.727	2.697	0.635
	O6	**2.4464**	**1.440**	0.372

A \ B	I2	I3	O6
I2		118.3	126.1
I3	Bond angles A-Zn-B		114.7
O6			

Supplementary Table 3. ***Ab initio*** **calculation model of** [ZnI_2] and PEG300 in ZnI_2 electrolytes. During the bonding process, one Iodine ion departs from the main structure.

	R_{int} (Ω)	Error (±)
$ZnSO_4$	850	22
$ZnSO_4$ + 5% PEG300	245	14
$ZnCl_2$	225	15
$ZnCl_2$ + 5% PEG300	180	12
$ZnBr_2$	75	10
$ZnBr_2$ + 5% PEG300	33	5
ZnI_2	8	6
ZnI_2 + 5% PEG300	5	5

Supplementary Table 4. Interfacial resistance R_{int} (Ω) fitted from the data and equivalent circuit model on Figure 4 and Supplementary Figure 12. The measurements were all performed in Zn||Zn symmetric cells.

Supplementary Note 1. Analysis on the electroconvection region of the current-voltage (i-V) curve.

Supplementary Figure 14 reports the current-voltage (i-V) curve for all electrolytes used in the study. The figure shows that the limiting current and voltage stability window (EVSW) ΔV are impacted by the PEG oligomer additive.[15,16] For Zn halide + PEG electrolytes, the limiting currents are higher than those in $ZnSO_4$ + PEG electrolyte, which we attribute to more resistive interfaces formed in the latter electrolytes.[13] At voltages above a critical value electroconvection will be triggered, which produces mixing in the electrolyte, enabling higher currents (over limiting conductance) above the diffusion limit for the electrolyte. The results in **Supplementary Figure 14** show that while over-limiting conductance is not eliminated by the polymer, its onset is delayed to higher voltages, effectively extending the voltage stability windows ΔV for the electrolytes. I-V curves measured at different salt concentrations provide a more precise understanding of the effect of the ion-oligomer adsorption intensity and EVSW ΔV (**Supplementary Figure 14**). Based on **Figure 2**, the bonding effect between solvated Zn ions and PEG300 is weak at low concentration (<0.1M) since the ^{1}H shift of PEG300 is negligible at low concentration. As shown in **Supplementary Figure 14c**, at 0.05M $ZnSO_4$ and ZnX_2 + 5 wt% PEG300 electrolytes, the EVSW ΔV value is close to each other. Here, the term weak bonding is used to indicate that the ions do not influence the polymer adsorption process, implying that the electroconvection suppression performance is the same. However, as we increase the concentration to 0.5M (**Supplementary Figure 14 d.e.f**), the ΔV is larger in Zn halide +PEG300 electrolytes than in $ZnSO_4$ + PEG300 electrolytes. At much higher concentration salts (1M), a small ΔV is observed in these four pure Zn salt electrolytes (**Supplementary Figure 14 g.h**), which should result from the cation adsorption, while much larger EVSW ΔV is shown in Zn halide + PEG300 electrolytes (**Supplementary Figure 14i**), indicating the expected ion electroconvection suppression effect here. However, the ΔV disappeared in $ZnSO_4$ + PEG300 electrolytes, which is because compared with SO_4^{2-}, the high entropy of PEG300 enables the surface separation of ion-oligomer adsorption layer which stresses the importance of stronger interfacial adsorption. Taken together of all ΔV of these four electrolytes, a much stronger control to the ion electroconvection is proved in Zn halide + PEG electrolytes, which originate from the intense interfacial ion-oligomer adsorption.

References:

1 D'Angelo, P., Zitolo, A., Ceccacci, F., Caminiti, R. & Aquilanti, G. Structural characterization of zinc(II) chloride in aqueous solution and in the protic ionic liquid ethyl ammonium nitrate by x-ray absorption spectroscopy. *J Chem Phys* **135**, 154509, doi:10.1063/1.3653939 (2011).

2 Gordon, M. S. & Schmidt., M. W. Advances in electronic structure theory: GAMESS a decade later In Theory and applications of computational chemistry. *Elsevier*, 1167-1189 (2005).

3 Schmidt, M. W. *et al.* General atomic and molecular electronic structure system. *Journal of computational chemistry* **14**, 1347-1363 (1993).

4 Pan, H. *et al.* Reversible aqueous zinc/manganese oxide energy storage from conversion reactions. *Nature Energy* **1**, doi:10.1038/nenergy.2016.39 (2016).

5 Cai, Z. *et al.* A Replacement Reaction Enabled Interdigitated Metal/Solid Electrolyte Architecture for Battery Cycling at 20 mA cm(-2) and 20 mAh cm(-2). *J Am Chem Soc* **143**, 3143-3152, doi:10.1021/jacs.0c11753 (2021).

6 Liang, P. *et al.* Highly Reversible Zn Anode Enabled by Controllable Formation of Nucleation Sites for Zn-Based Batteries. *Advanced Functional Materials* **30**, doi:10.1002/adfm.201908528 (2020).

7 Shen, C. *et al.* Graphene-Boosted, High-Performance Aqueous Zn-Ion Battery. *ACS Appl Mater Interfaces* **10**, 25446-25453, doi:10.1021/acsami.8b07781 (2018).

8 Yuksel, R., Buyukcakir, O., Seong, W. K. & Ruoff, R. S. Metal-Organic Framework Integrated Anodes for Aqueous Zinc-Ion Batteries. *Advanced Energy Materials* **10**, doi:10.1002/aenm.201904215 (2020).

9 Zeng, Y. *et al.* Dendrite-Free Zinc Deposition Induced by Multifunctional CNT Frameworks for Stable Flexible Zn-Ion Batteries. *Adv Mater* **31**, e1903675, doi:10.1002/adma.201903675 (2019).

10 Zhang, Q. *et al.* The Three-Dimensional Dendrite-Free Zinc Anode on a Copper Mesh with a Zinc-Oriented Polyacrylamide Electrolyte Additive. *Angew Chem Int Ed Engl* **58**, 15841-15847, doi:10.1002/anie.201907830 (2019).

11 Zhao, K. *et al.* Ultrathin Surface Coating Enables Stabilized Zinc Metal Anode. *Advanced Materials Interfaces* **5**, doi:10.1002/admi.201800848 (2018).

12 Zheng, J. *et al.* Reversible epitaxial electrodeposition of metals in battery anodes. *Science* **366,** (2019).

13 Jin, S. *et al.* Stabilizing Zinc Electrodeposition in a Battery Anode by Controlling Crystal Growth. *Small* **17**, e2101798, doi:10.1002/smll.202101798 (2021).

14 Cao, L. *et al.* Fluorinated interphase enables reversible aqueous zinc battery chemistries. *Nat Nanotechnol* **16**, 902-910, doi:10.1038/s41565-021-00905-4 (2021).

15 Tikekar, M. D., Li, G., Archer, L. A. & Koch, D. L. Electroconvection and Morphological Instabilities in Potentiostatic Electrodeposition across Liquid Electrolytes with Polymer Additives. *Journal of The Electrochemical Society* **165**, A3697-A3713, doi:10.1149/2.0271816jes (2018).

16 Wei, S. *et al.* Stabilizing electrochemical interfaces in viscoelastic liquid electrolytes. *Science advances* **4**, eaao6243 (2018).
